# DOT-1.1-dependent H3K79 methylation promotes normal meiotic progression and meiotic checkpoint function in *C*. *elegans*

**DOI:** 10.1371/journal.pgen.1009171

**Published:** 2020-10-26

**Authors:** Laura I. Lascarez-Lagunas, Esther Herruzo, Alla Grishok, Pedro A. San-Segundo, Mónica P. Colaiácovo

**Affiliations:** 1 Department of Genetics, Blavatnik Institute, Harvard Medical School, Boston, MA, United States of America; 2 Instituto de Biología Funcional y Genómica, Consejo Superior de Investigaciones Científicas and University of Salamanca, Salamanca, Spain; 3 Department of Biochemistry, Boston University School of Medicine, Boston, MA, United States of America; 4 Genome Science Institute, Boston University School of Medicine, Boston, MA, United States of America; University of California, Davis, UNITED STATES

## Abstract

Epigenetic modifiers are emerging as important regulators of the genome. However, how they regulate specific processes during meiosis is not well understood. Methylation of H3K79 by the histone methyltransferase Dot1 has been shown to be involved in the maintenance of genomic stability in various organisms. In *S*. *cerevisiae*, Dot1 modulates the meiotic checkpoint response triggered by synapsis and/or recombination defects by promoting Hop1-dependent Mek1 activation and Hop1 distribution along unsynapsed meiotic chromosomes, at least in part, by regulating Pch2 localization. However, how this protein regulates meiosis in metazoans is unknown. Here, we describe the effects of H3K79me depletion via analysis of *dot-1*.*1* or *zfp-1* mutants during meiosis in *Caenorhabditis elegans*. We observed decreased fertility and increased embryonic lethality in *dot-1*.*1* mutants suggesting meiotic dysfunction. We show that DOT-1.1 plays a role in the regulation of pairing, synapsis and recombination in the worm. Furthermore, we demonstrate that DOT-1.1 is an important regulator of mechanisms surveilling chromosome synapsis during meiosis. In sum, our results reveal that regulation of H3K79me plays an important role in coordinating events during meiosis in *C*. *elegans*.

## Author summary

Sexual reproduction relies on the production of sperm and eggs by the specialized cell division program called meiosis. Errors during meiosis often lead to miscarriages, infertility, and birth defects. Therefore, understanding the mechanisms regulating meiosis has major implications for human health. Chromatin modifiers have emerged as important regulators of a wide-range of cellular processes. However, despite their importance, their roles during meiosis remain poorly understood. Here, we show that *C*. *elegans* DOT-1.1, a conserved histone methyltransferase that catalyzes histone H3 lysine 79 (H3K79) methylation, regulates chromosome pairing, synapsis and recombination, thereby promoting normal meiotic progression and chromosome segregation. Moreover, we show that DOT-1.1 promotes a meiotic checkpoint function set in place to survey chromosome synapsis through a mechanism that appears to be distinct from the one described in budding yeast. Altogether, our study reveals a link between the regulation of H3K79me and the normal progression of meiosis.

## Introduction

Meiosis is an essential cell division program for all sexually reproducing organisms. It halves the genome’s content by following one round of DNA replication with two successive rounds of cell division, meiosis I and II, to generate haploid gametes (i.e. sperm and oocytes). A series of well-orchestrated events ensure accurate homologous chromosome segregation at meiosis I while preserving sister chromatid associations until meiosis II [1]. Namely, homologs have to pair, synapse and recombine. Errors in any of these processes can lead to the formation of aneuploid gametes, which can result in birth defects such as Down syndrome, miscarriages and infertility in humans [2]. While many of the proteins required for achieving homologous pairing, synapsis and recombination are known, far less is understood about how dynamic changes in the chromatin landscape affect these processes during meiosis.

Alterations in the chromatin landscape are mediated in part by post-translational modifications of histones which form octamers wrapped by DNA (one H3/H4 heterotetramer and two H2A/H2B dimers) to form the building blocks of chromatin, the nucleosomes [3–6]. Post-translational modifications of histones play an important role in the establishment and maintenance of gene expression, and covalent histone modifications influence chromatin structure and function directly or indirectly through the recruitment of effector proteins to specific chromatin domains [7–9].

Histones can undergo several types of modifications including acetylation, phosphorylation and methylation. One of these histone modifications is the methylation of H3K79 (hereafter H3K79me) by the histone methyltransferase Dot1 (disruptor of telomeric silencing in yeast [10–12]), which has been reported to be involved in the maintenance of genomic stability in various organisms [13–15]. Dot1 is a methyltransferase that catalyzes mono-, di- and trimethylation (me1, me2 and me3, respectively) of histone H3K79 [12,16]. A demethylase for this histone mark has not been identified so far. Dot1 is an evolutionarily conserved protein that regulates diverse cellular processes, such as development, reprogramming, differentiation, and proliferation [17–20]. During meiosis in yeast, Dot1 modulates the meiotic checkpoint response induced in the *zip1* mutant lacking a major component of the central region of the synaptonemal complex (SC). Dot1 promotes Hop1-dependent Mek1 activation and Hop1 distribution along unsynapsed meiotic chromosomes [18]. Several lines of evidence suggest that Dot1 regulates this checkpoint, at least in part, by defining proper Pch2 chromosomal distribution [18,21]. In mammals, *Dot1L* (Dot1 (yeast)-Like) is essential for embryo viability [22], and enhanced activity of DOT1L enzyme is observed in mixed lineage leukemia (MLL) [15]. The *C*. *elegans* genome encodes five putative methyltransferases of the Dot1 family [11], among which DOT-1.1 has been shown, through computational and experimental analysis, to be the homolog of mammalian DOT1L [19,23]. Although cytological analyses of DOT1L and H3K79me distribution in mouse spermatocytes are suggestive of a functional implication for this histone modification in mammalian meiosis [24], the roles of DOT1L and regulation of H3K79 methylation during meiosis had not been directly examined in a metazoan.

Despite its importance, the impact of the chromatin environment during meiotic progression has been poorly studied. Here we describe the roles of DOT-1.1 and H3K79me in the germline of *C*. *elegans*. Analysis of *dot-1*.*1* mutants revealed that DOT-1.1 regulates the levels of H3K79me in the germline. *dot-1*.*1* mutants show a decreased brood size and increased embryonic lethality, which may result from meiotic defects that lead to errors in chromosome segregation and the formation of aneuploid gametes. This is further supported by the presence of an extended leptotene/zygotene (transition zone) region, impaired homologous pairing, nuclei with incomplete synapsis, a decreased number of DNA double-strand breaks (DSBs), an altered number of crossovers (COs) and chromosome morphology defects in oocytes at diakinesis observed in *dot-1*.*1* mutants. Importantly, the striking extension of CHK-2 activity observed in the chromosome synapsis defective *syp-1* mutant is reduced in *dot-1*.*1; syp-1* double mutants suggesting that DOT-1.1 may regulate the activation/establishment of the synapsis checkpoint in *C*. *elegans*. However, unlike in yeast, the mechanism of such regulation is independent of the chromosomal localization of PCH-2. Altogether, our study reveals a role for DOT-1.1 in the regulation of key meiotic processes including surveillance mechanisms therefore linking regulation of H3K79me to normal progression of meiotic events in *C*. *elegans*.

## Results

### DOT-1.1 regulates the levels of H3K79me in the germline

Dot1 and its homologs appear to be solely responsible for H3K79 methylation since knockout of Dot1 in yeast, flies, and mice result in complete loss of H3K79 methylation [10,22,25]. The yeast protein Dot1 and its human homolog, DOT1L, are able to catalyze mono-, di-, and trimethylation in a non processive manner [16,26]. In *C*. *elegans*, levels of H3K79me2 are almost absent in whole worm extracts from L3 stage *dot-1*.*1; ced-3* mutants [23]. To determine the role of DOT-1.1 and H3K79me regulation in the germline, our analyses were done using a *dot-1*.*1(knu339); ced-3(n1286)* double mutant, unless indicated otherwise. *dot-1*.*1(knu339)* carries a deletion of exons 1 through 4 in the *dot-1*.*1* gene locus and has been described as a null mutant [23]. Since *dot-1*.*1* null mutants do not survive due to massive apoptosis, with the worms dying as arrested larvae [27], we circumvented this with a mutation in *ced-3* that encodes for a homolog of mammalian caspase-3 as in [23].

**Fig 1:**
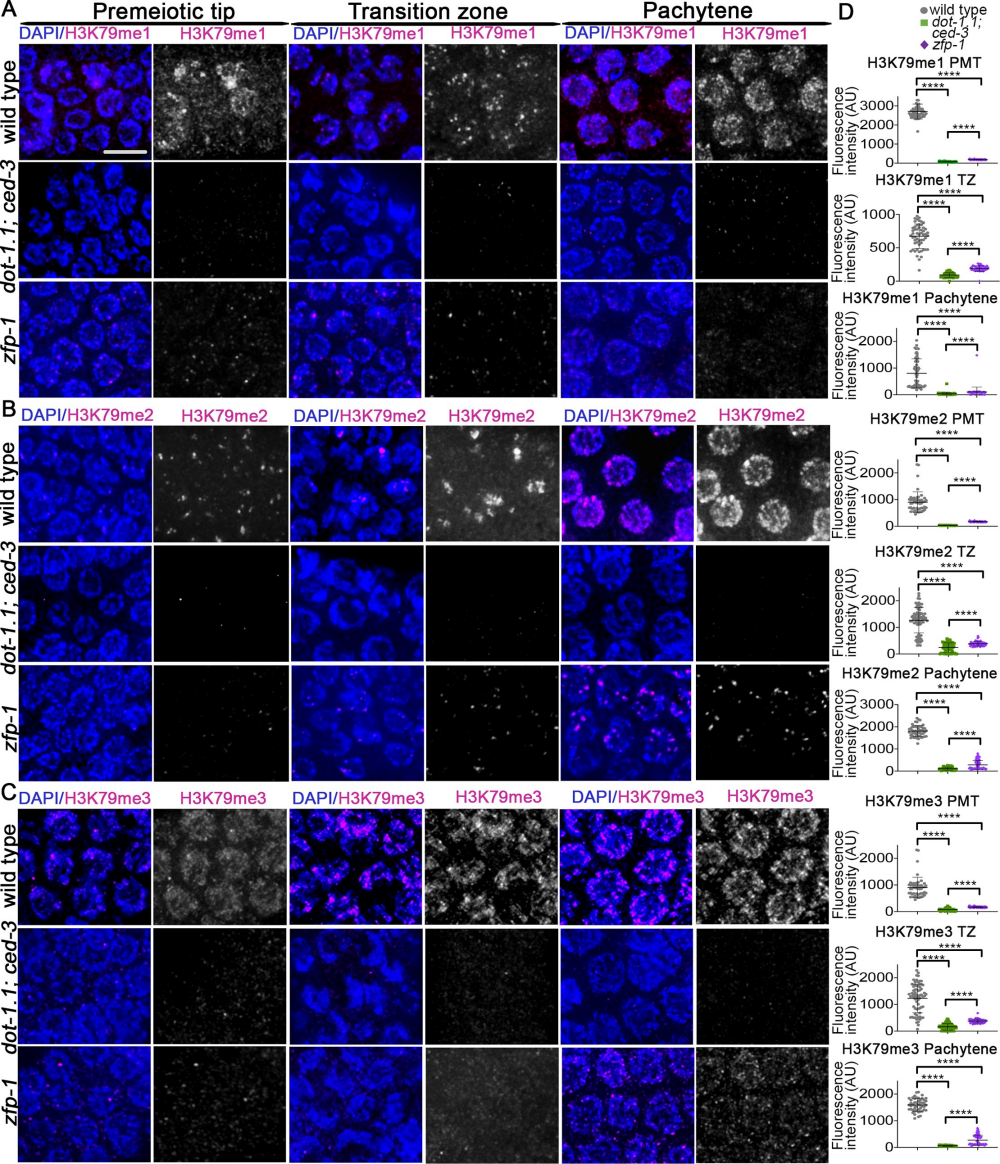
DOT-1.1 and ZFP-1 are required for normal chromatin-associated H3K79me1,-2,-3 signal in the *C*. *elegans* germline. High-resolution images of germline nuclei from the indicated genotypes co-stained with DAPI (blue) and **(A)** H3K79me1 (magenta), **(B)** H3K79me2 (magenta), and **(C)** H3K79me3 (magenta). In wild-type worms, H3K79me1, -2 and -3 signal is associated with the chromatin through the entire gonad. However, in *dot-1*.*1; ced-3* and, less dramatically, in the *zfp-1* mutant, this signal is decreased. Premeiotic tip (zone 2), transition zone (zone 3) and pachytene (zone 5) nuclei are shown. Scale bar, 5 μm. **(D)** Quantification of fluorescence intensity detected for each genotype at the indicated zones in the gonads. Fluorescence intensity was sampled from a minimum of 68 nuclei from at least 6 gonads for each zone and quantitated using ImageJ software. PMT, Premeiotic tip; TZ, transition zone. **** P<0.0001 by the two-tailed Mann-Whitney test, 95% C.I.

To analyze the pattern of H3K79me in the germline we stained whole-mounted gonads of wild-type and *dot-1*.*1; ced-3* worms with antibodies against H3K79 mono-, di- and trimethylation. We observed that H3K79me signal is present throughout the gonad starting at the premeiotic tip and extending through late pachytene (Fig 1). H3K79me1 and H3K79me2 signals were observed as punctae or large aggregates in nuclei at the premeiotic tip and transition zone, and more uniformly distributed along the chromosomes in pachytene nuclei (Fig 1A and 1B), whereas H3K79me3 signal was more evenly distributed through the chromosomes from premeiotic tip through pachytene (Fig 1C). Co-staining with antibodies against H3K79 mono-, di- and trimethylation and a pan acetylation antibody (AcK), which allows for identification of the X chromosomes as they exhibit greatly decreased histone acetylation compared to the autosomes during early meiotic prophase [28], revealed even distribution of H3K79 mono-, di- and trimethylation signal on autosomes and the X chromosome (S1 Fig). Finally, analysis of H3K79me in *dot-1*.*1; ced-3* worms revealed that H3K79 mono-, di- and trimethylation signals are significantly decreased throughout the gonad compared to wild type, supporting a major role for DOT-1.1 in regulating the levels of H3K79me in the germline in *C*. *elegans* (Fig 1A–1D).

### *dot-1*.*1* mutant worms exhibit sterility, increased embryonic lethality and altered germline chromosome morphogenesis

**Fig 2:**
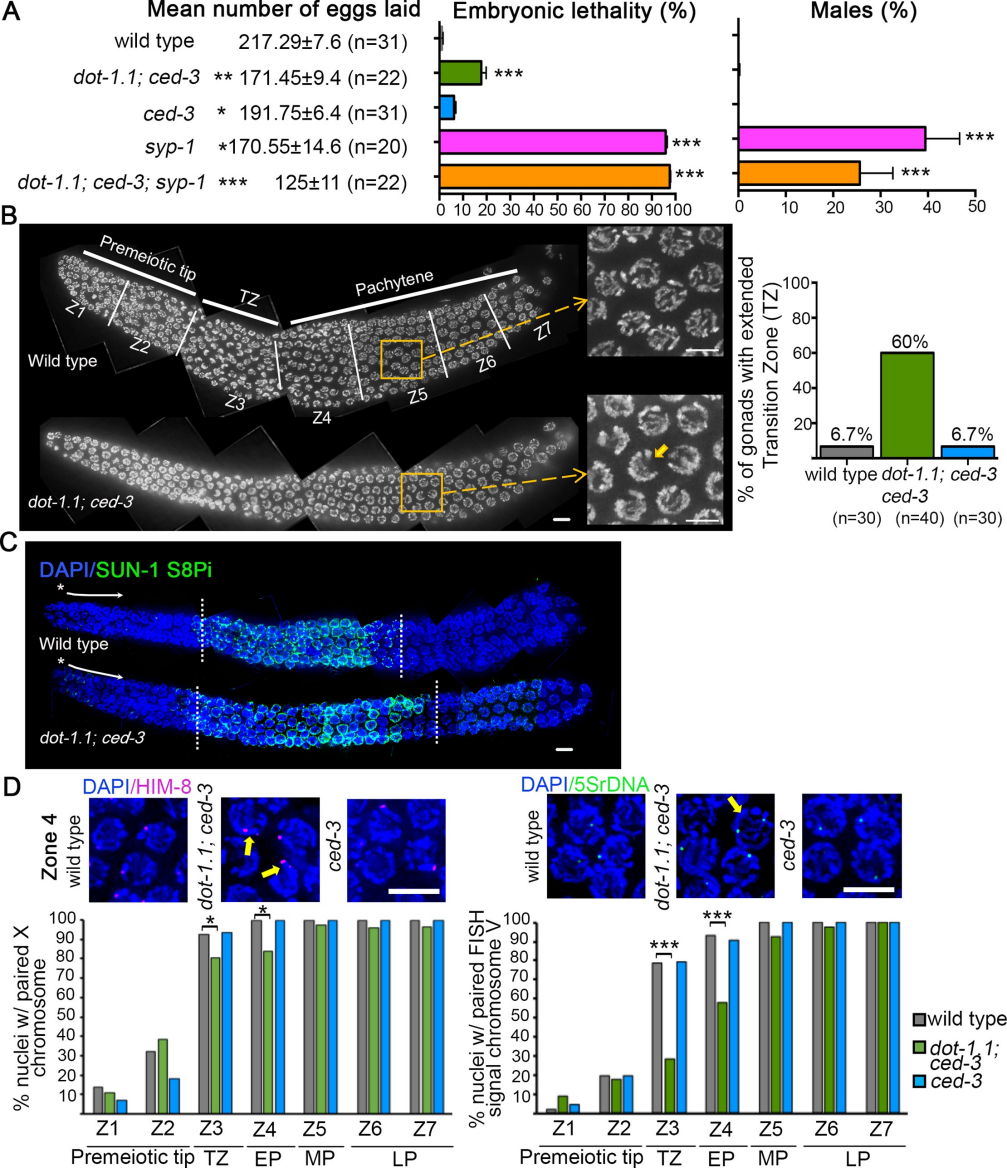
*dot-1*.*1* mutant worms exhibit increased embryonic lethality, sterility and defects in chromosomal organization in the germline. **(A)** The mean number of eggs laid (brood size) as well as the percentage of embryonic lethality and males are shown for the indicated mutants and compared to wild type. Error bars represent SEM. *P<0.013, **P<0.0025, and ***P<0.00025 by the two-tailed Mann-Whitney test, 95% C.I., with Bonferroni correction for multiple comparisons. n = number of worms analyzed. **(B)** Left, high-resolution images of whole mounted gonads stained with DAPI from wild-type and *dot-1*.*1; ced-3* mutant animals oriented from left to right. The different stages of meiotic prophase are indicated above the gonads and the seven equally-sized zones scored (Z1-Z7) are delimited by vertical white lines. Nuclei in Z1 and Z2 are undergoing mitosis. They enter meiosis at Z3, referred to as the transition zone (TZ), which corresponds to the leptotene/zygotene stages. Nuclei then proceed through pachytene (Z4-Z7). Insets show the presence of transition zone-like nuclei (small arrow) in mid-pachytene for *dot-1*.*1; ced-3* mutant worms. Right, histogram indicates percentage of gonads exhibiting nuclei with chromatin in a leptotene/zygotene-like organization in early and mid-pachytene stages (zones 4 and 5). Data was obtained from 4 to 6 independent biological repeats for wild type (n = 30), *dot-1*.*1; ced-3* (n = 40) and *ced-3* (n = 30). n = number of gonads scored. Scale bars, 5 μm. **(C)** Whole mounted gonads from wild-type and *dot-1*.*1; ced-3* worms co-immunostained for SUN-1(pS8) (green) and DAPI (blue). *dot-1*.*1; ced-3* worms show SUN-1 positive nuclei persisting into mid-pachytene in prophase I compared to wild type, suggesting defects in meiotic progression. Asterisks indicate the premeiotic tip and white arrows show the direction in which nuclei move proximally in the germline (meiotic progression). Hatched lines demarcate the first (left) and the last (right) row with nuclei showing SUN-1 (pS8) signal. At least 30 gonads from two independent biological repeats were analyzed for each genotype. Scale bars, 5 μm. **(D)** Top left, high-resolution images of early pachytene nuclei (zone 4) co-stained with HIM-8 (magenta) and DAPI (blue). Yellow arrows indicate nuclei with unpaired HIM-8 signal. Scale bar 5 μm. Bottom left, histogram representing the percentage of nuclei with paired HIM-8 signals scored at different zones along the germline in wild type, *dot-1*.*1; ced-3* and *ced-3*. *P<0.025, Fisher’s exact test after Bonferroni correction. Top right, high-resolution images of early pachytene nuclei (zone 4) stained with DAPI and hybridized with a FISH probe recognizing the 5S rDNA locus on chromosome V (green). Yellow arrow indicates a nucleus with unpaired FISH signal. Scale bar 5 μm. Right bottom, histogram representing the percentage of nuclei with paired FISH signal (5S rDNA) scored at different zones along the germline in wild type, *dot-1*.*1; ced-3* and *ced-3*. ***P<0.0005, Fisher’s exact test after Bonferroni correction.

To determine the role of DOT-1.1 during meiotic prophase we assessed whether *dot-1*.*1; ced-3* mutants exhibit a decrease in the number of eggs laid (brood size), which is indicative of sterility, an increase in the number of unhatched eggs (embryonic lethality; Emb) and a high incidence of males (Him) among the surviving progeny, which are phenotypes suggestive of impaired meiotic chromosome segregation (although Emb can also result from defects in embryonic development) (Fig 2). While a 21% reduction in the mean number of eggs laid on plates was observed in *dot-1*.*1; ced-3* mutants compared to wild type (171.45±9.42 and 217.29±7.66, respectively; P<0.0025 by the two-tailed Mann-Whitney test, 95% C.I.), a 12% reduction was observed in the *ced-3* single mutant (191.75±6.44; P<0.013) (Fig 2A). However, the decrease in the number of eggs laid by *dot-1*.*1; ced-3* mutants compared to the *ced-3* single mutant is also significant (171.45±9.42 and 191.75±6.44; P<0.013). We also observed significantly increased embryonic lethality, but not a high incidence of males, in *dot-1*.*1; ced-3* mutants compared to wild type (17.8% and 1%, respectively; P<0.00025) (Fig 2A). In addition, a mild and not significant increase in embryonic lethality was observed in *ced-3* single mutants compared to wild type (6.2% and 1%, respectively; P = 0.015), however there is a significant difference between the embryonic lethality observed in *dot-1*.*1; ced-3* and the *ced-3* single mutant (17.8% and 6.2%, respectively; P<0.0025). These combined results suggest that most of the sterility and increased embryonic lethality observed in *dot-1*.*1; ced-3* results from the *dot-1*.*1* mutation itself.

To explore whether the increased sterility and embryonic lethality is due, at least in part, to defects occurring during meiosis, we examined DAPI-stained gonads from wild-type, *dot-1*.*1; ced-3* and *ced-3* mutant worms. In the *C*. *elegans* germline, nuclei are organized in a spatial-temporal gradient thereby facilitating the identification of alterations in chromosome organization at specific meiotic stages [29]. We observed an increase in the number of gonads with nuclei exhibiting chromatin in a leptotene/zygotene-like organization (crescent shape configuration) at the early and mid-pachytene stages (zones 4–5) in *dot-1*.*1; ced-3* mutants compared with either wild type or *ced-3* alone (60%, n = 40, 6.7%, n = 30 and 6.7%, n = 30 respectively) revealing an extended transition zone in the absence of DOT-1.1 (Fig 2B). To further examine if either entry into meiosis or meiotic progression might be affected, we examined the localization of phosphorylated SUN-1 (pS8), which forms aggregates at the nuclear envelope primarily during leptotene/zygotene and then becomes weaker and dispersed during early to mid-pachytene in wild type ([30] and Fig 2C). In *dot-1*.*1; ced-3* worms, SUN-1 (pS8) signal starts to appear in leptotene/zygotene, similar to wild type, suggesting that entry into meiosis is normal in this mutant. However, the presence of SUN-1 (pS8)-positive nuclei in *dot-1*.*1; ced-3* germlines extends into mid-pachytene, while they are no longer present at that stage in wild type, suggesting problems with meiotic progression (Fig 2C). Taken together, these data suggest that DOT-1.1 is required for normal meiotic chromosome morphogenesis and segregation, without ruling out a possible contribution for DOT-1.1 to embryonic development.

### DOT-1.1 is required for normal progression of homologous pairing and SC assembly

The persistence of nuclei with a transition zone morphology at the early and mid-pachytene stages (zones 4–5) has been previously associated with a delay in chromosome pairing and with defects in SC formation [31]. To examine homologous pairing, we divided the germlines of *dot-1*.*1; ced-3*, *ced-3* and wild-type hermaphrodites into seven zones of equal size and evaluated the pairing frequencies for the pairing center end (a *cis*-acting region implicated in homolog recognition) of the X-chromosome, visualized by localization of the zinc finger protein HIM-8 to that region [32], and for a more internal region of chromosome V (5S rDNA locus) by fluorescence in situ hybridization (FISH) [33]. Chromosomes were scored as paired when HIM-8 or 5S rDNA foci were ≤ 0.75 μm apart. In wild-type and *ced-3* hermaphrodites, homologous pairing for both the X chromosome and chromosome V was observed initiating at transition zone (zone 3; Fig 2B and 2D) (there is a background level of association between homologs in the premeiotic tip, as previously reported; [32]), with at least 92.8% of nuclei exhibiting homologous pairing by early-pachytene and ~100% by mid-pachytene (zones 4 and 5; Fig 2B and 2D). In contrast, in *dot-1*.*1; ced-3* hermaphrodites, we observed a delay in pairing as shown by the significantly lower levels of nuclei with paired HIM-8 or 5S rDNA signal starting at transition zone and persisting into early pachytene (zones 3 through 4, Fig 2D; P<0.025 and P<0.0005, respectively, by the Fisher’s exact test). While chromosome V was observed paired in 100% of nuclei by late pachytene (zone 7), between 95% to 97% of nuclei exhibited paired X chromosomes from mid to late pachytene (zones 5 through 7), which was not significantly different from wild-type worms. Nevertheless, there were a few nuclei exhibiting unpaired HIM-8 signal until zone 7 in *dot-1*.*1; ced-3* mutants.

**Fig 3:**
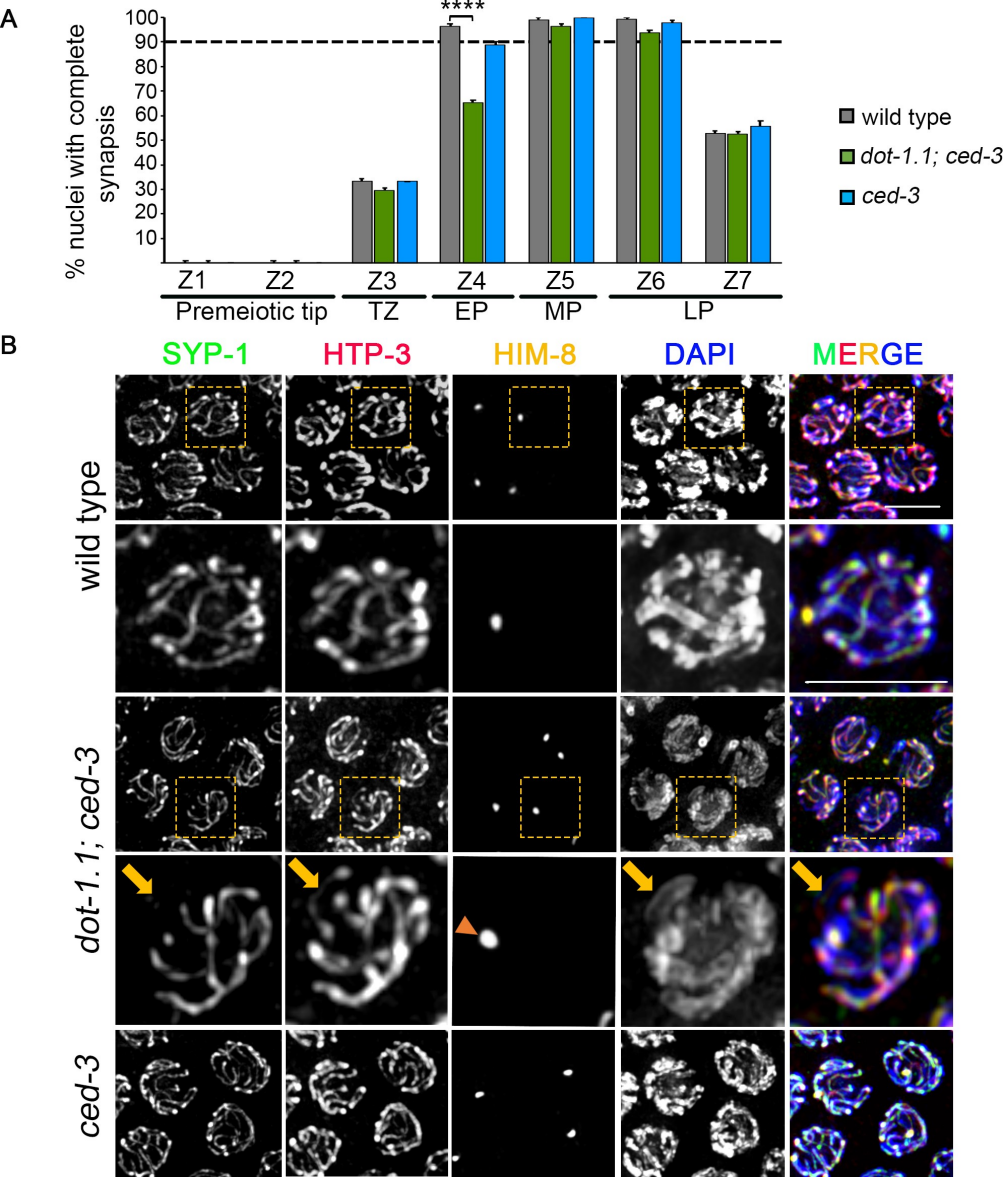
SYP-1 loading on chromosomes is affected in *dot-1*.*1* mutants. **(A)** Histogram indicating the percentage of nuclei that exhibit complete synapsis as a function of meiotic progression in wild-type, *dot-1*.*1; ced-3* and *ced-3* worms. Nuclei showing complete overlapping signal of the lateral element component HTP-3 and the central region component SYP-1 along all chromosomes were considered as nuclei with complete synapsis. Between 5 to 7 gonads from three biological repeats were scored for wild type (n = 312), *dot-1*.*1; ced-3* (n = 419) and *ced-3* (n = 320); n = number of nuclei scored. Dashed horizontal line indicates 90% for better visualization of minor differences in zones 5 and 6. **(B)** High-resolution images of mid-pachytene nuclei co-immunostained with SYP-1 (green), HTP-3 (red), HIM-8 (yellow) and DAPI (blue). Dashed boxes indicate the nuclei shown at higher magnification for wild type and *dot-1*.*1; ced-3* mutants. Unlike wild-type and *ced-3* mutant worms, 9.8% of nuclei (41/419) in *dot-1*.*1; ced-3* mutants exhibited DAPI-stained regions lacking SYP-1 signal at the pachytene stage. 36.6% (15/41) of these nuclei exhibited discontinuities in HTP-3 signal and in 61% (25/41) the unsynapsed chromosome (arrow) corresponded to the X chromosome as determined by presence of HIM-8 signal (arrowhead). TZ, transition zone; EP, early pachytene; MP, mid-pachytene; LP, late pachytene. Scale bars, 5 μm.

To examine SC assembly, we co-stained whole-mounted gonads from wild type, *dot-1*.*1; ced-3* and *ced-3* mutants with antibodies against HTP-3, a lateral element component of the SC [34,35], and SYP-1, a central region component of the SC [31], and scored the percentage of nuclei with complete synapsis as a function of meiotic progression (Fig 3). In wild-type worms, initiation of SC assembly, defined by the presence of short patches of central region components on chromosomes with lateral element proteins fully loaded throughout the full length of the chromosomes, was first observed at transition zone (zone 3), and 96% of nuclei had completed SC assembly, based on co-localization of HTP-3 and SYP-1 between all chromosome pairs, by early pachytene (zone 4). *dot-1; ced-3* worms also initiated SC assembly at transition zone, but only 65% of nuclei had completed SC assembly by early pachytene indicating a delay in SC assembly compared to wild type (P<0.0001, Fisher’s exact test) (Fig 3A). Such defect seems to be specific to *dot-1*.*1* since it was not observed in the *ced-3* single mutant. We observed similar levels of SC disassembly between wild-type and *dot-1*.*1; ced-3* mutant worms (Fig 3A; zone 7). More detailed analysis showed that 10% of the combined nuclei from mid to late pachytene (zones 5 and 6; n = 419) in *dot-1*.*1; ced-3* mutants did not have SYP-1 signal in at least one chromosome compared to 0.64% (n = 312) and 0.63% (n = 320) observed in wild type and the *ced-3* single mutant, respectively. From those, 36.6% (15/41) also lacked HTP-3 signal explaining the absence of SYP-1 since proper assembly of the SC depends on the normal formation of axes [35]. The remaining nuclei, 63.4% (26/41), lacked SYP-1 signal although HTP-3 signal was not altered (Fig 3B), suggesting that DOT-1.1 is implicated in the regulation of SYP-1 loading itself. Furthermore, the absence of SYP-1 was mainly restricted to one chromosome in each nucleus. Co-immunostaining for SYP-1, HTP-3 and HIM-8 revealed that 61% of the chromosomes without SYP-1 signal (25/41) were positive for HIM-8, indicating that the X chromosome is more dependent on DOT-1.1 for SYP-1 loading compared to the autosomes (Fig 3B).

### DNA double-strand break formation is impaired in *dot-1*.*1* mutants

**Fig 4:**
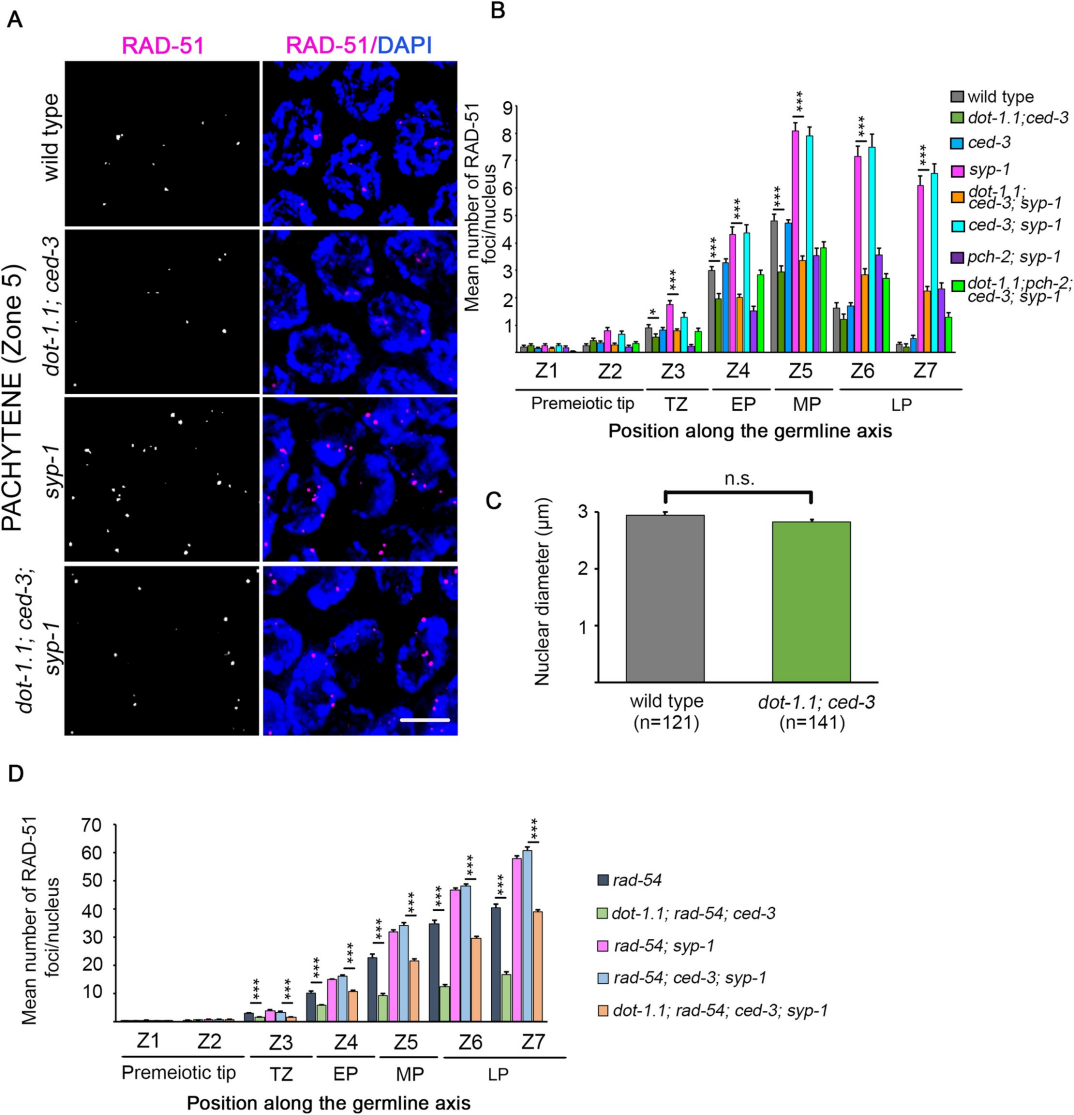
DSB formation is altered in *dot-1*.*1* mutant worms. **(A)** High-resolution images representative of mid-pachytene nuclei (zone 5) immunostained for RAD-51 (magenta) and co-stained with DAPI (blue). Scale bar, 5 μm. **(B)** Histogram shows the mean number of RAD-51 foci/nucleus (y-axis) scored along each zone in the germlines (x-axis) of the indicated genotypes. Between 4 and 6 gonads were scored per genotype. A significant decrease in levels of RAD-51 foci were observed for zones 4 to 7 in *dot-1*.*1; ced-3* germlines compared to wild-type and in *dot-1*.*1; ced-3; syp-1* germlines compared to *syp-1*. Error bars represent SEM from technical repeats for each of two to three biological replicates (*P<0.007 and ***P<0.0001 by the two-tailed Mann-Whitney test, 95% C.I., after Bonferroni correction). **(C)** Graph depicting the mean nuclear diameter observed for premeiotic tip nuclei for the indicated genotypes. n = number of nuclei scored. n.s. = not significant. **(D)** Histogram shows the mean number of RAD-51 foci/nucleus for each zone along the germlines of *rad-54* combinatorial mutants. Decreased levels of DSBs for *dot-1*.*1; rad-54; ced-3* compared to *rad-54*, and for *dot-1*.*1; rad-54; ced-3; syp-1* compared to *rad-54; ced-3; syp-1*, were observed beginning in transition zone (zone 3) and persisting until late pachytene (zone 7). Error bars represent SEM for technical repeats from two biological repeats (***P<0.0001 by the two-tailed Mann-Whitney test, 95% C.I., after Bonferroni correction). TZ, transition zone; EP, early pachytene; MP, mid- pachytene; LP, late pachytene.

Since impaired homologous pairing and SC assembly can lead to defects in meiotic recombination, we assessed meiotic DSB repair progression by quantifying the levels of RAD-51 foci on immunostained whole-mounted gonads in wild type and *dot-1*.*1; ced-3* mutants (Fig 4, S1 and S2 Tables). RAD-51 binds to 3’ ssDNA ends at resected DSBs to promote strand invasion/exchange during DSB repair [36], and in *C*. *elegans*, RAD-51 foci on chromosomes indicate sites undergoing DSB repair [37]. We scored the number of RAD-51 foci per nucleus throughout the germline. In wild-type and *ced-3* mutant gonads, very low levels of RAD-51 foci were observed at the premeiotic tip (zones 1–2). RAD-51 foci levels start to increase upon entrance into meiosis at transition zone (zone 3), peak by mid-pachytene (zone 5) and then decrease by late pachytene (zones 6 and 7) as DSB repair is completed (Fig 4A and 4B). Similar to wild type, very low levels of RAD-51 foci were observed at the premeiotic tip in *dot-1*.*1; ced-3* worms suggesting replication is not affected in this mutant. This is further supported by the similar nuclear diameters measured for premeiotic tip nuclei in both wild-type and *dot-1*.*1; ced-3* germlines, given that S-phase arrest would have resulted in increased nuclear diameters in that region (Fig 4C and [38]). In contrast to wild type, *dot-1*.*1; ced-3* mutants showed significantly lower levels of RAD-51 foci in meiotic nuclei (zones 3–7). The lower levels of RAD-51 foci in *dot-1*.*1; ced-3* mutants could either be due to a reduction in the levels of DSB formation or to a faster turnover/repair of DSBs. To distinguish between these possibilities, we assayed RAD-51 foci in *rad-54* and *dot-1*.*1; rad-54; ced-3* triple mutants (Fig 4D, S1 and S2 Tables) given that a mutation in *rad-54* prevents the removal of RAD-51 from repair intermediates and stalls the progression of meiotic recombination, essentially “trapping” DSB-bound RAD-51 and allowing for quantification of the total number of DSBs [39]. *dot-1*.*1; rad-54; ced-3* mutants showed a significant decrease in the levels of RAD-51 foci in nuclei from leptotene/zygotene to late-pachytene stages compared to *rad-54* single mutants (zones 3 to 7; P<0.00025; Fig 4D) suggesting that DOT-1.1 may regulate levels of DSB formation.

### Levels of crossover formation are altered in *dot-1*.*1* mutants

**Fig 5:**
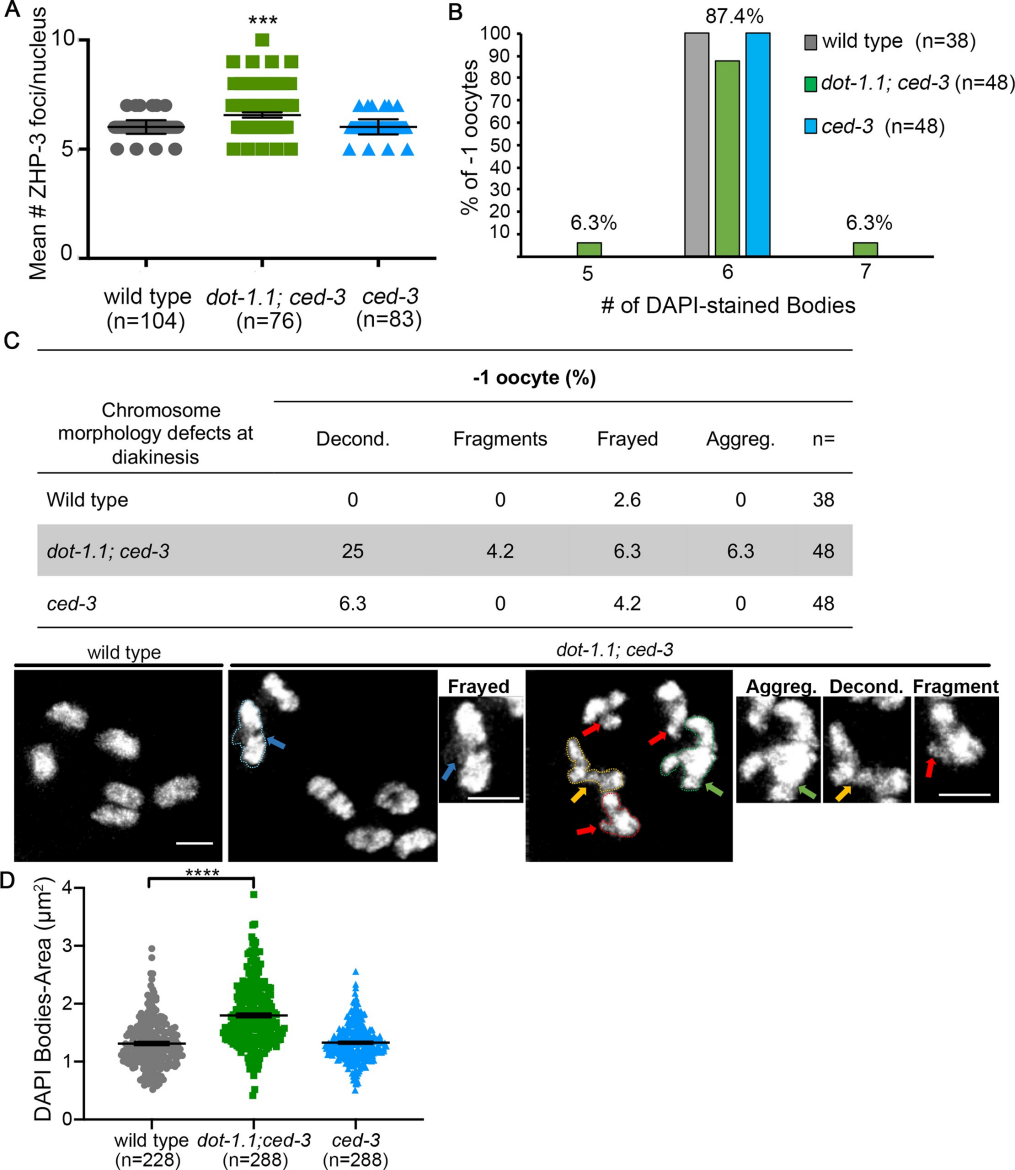
Altered crossover formation in *dot-1*.*1* mutants leads to bivalent morphology defects. **(A)** Histogram shows the mean number of ZHP-3 foci scored for each indicated genotype. Foci were quantified in late pachytene when six ZHP-3 foci per nucleus are clearly detected in wild type representing six COs (one per homolog pair). The number of nuclei scored for wild type, *dot-1*.*1; ced-3* and *ced-3* were n = 104, 76 and 83, respectively (from at least 5 gonads each from two biological repeats). Error bars represent SD (***P<0.001 by the two-tailed Mann-Whitney test, 95% C.I.). **(B)** Quantification of the number of DAPI-stained bodies observed in the -1 oocytes for the indicated genotypes. Wild-type and *ced-3* worms show 6 DAPI-stained bodies which correspond to 6 pairs of homologs. In contrast, oocytes with either 5 or 7 DAPI-stained bodies were detected in *dot-1*.*1; ced-3* mutant worms. The number of nuclei scored for wild type, *dot-1*.*1; ced-3* and *ced-3* were n = 38, 48 and 48, respectively from three biological repeats. **(C)** Top, table shows the quantification of the percentage of -1 oocytes at diakinesis displaying each one of the indicated defects in chromosome morphology. n = number of -1 oocytes scored. Decond. = decondensation. Aggreg. = aggregates. Bottom, representative high-resolution images of DAPI-stained bodies observed in -1 oocytes at diakinesis exhibiting either normal morphology (wild type) or defects including evidence of frayed chromosomes (blue arrows), aggregates (green arrows), chromosome decondensation (yellow arrows) and chromosome fragments (red arrows) in *dot-1*.*1; ced-3* mutants. Insets are higher magnification images of the DAPI-stained chromosomes traced with colored dotted lines corresponding to the colored arrows for specific defects. Scoring was done for three biological repeats. Scale bar, 2 μm. **(D)** Histogram shows the mean area measured for individual DAPI-stained bodies in -1 oocytes for the indicated genotypes. n = number of DAPI bodies scored. ****P<0.0001 by the two-tailed Mann-Whitney test, 95% C.I.

The number and distribution of crossovers (COs) along each pair of homologous chromosomes are tightly regulated throughout species [1]. This is particularly evident in *C*. *elegans* where only one CO occurs per homolog pair [40]. Given the alterations in DSB repair progression and decreased levels of DSB formation observed in *dot-1*.*1; ced-3* mutants, we used ZHP-3, the ortholog of budding yeast Zip3 and mammalian RNF212 [41,42], as a marker to quantify the number of sites designated to be repaired as COs in wild type, *dot-1*.*1; ced-3* and *ced-3* mutants (Fig 5A). In wild-type and *ced-3* hermaphrodites, a mean of 6 ZHP-3 foci per nucleus (n = 104 and 83, respectively) was observed by late pachytene, corresponding to one ZHP-3 focus for each of the six pairs of homologs. However, a mean of 6.6 ZHP-3 foci per nucleus (n = 76) was detected in *dot-1*.*1; ced-3* germlines (P< 0.0001, by the two-tailed Mann-Whitney test, 95% C.I.; Fig 5A). Since there are fewer DSBs formed in *dot-1*.*1; ced-3* mutants, but the mean number of COs is increased, this suggests that CO interference is altered in the absence of DOT-1.1.

To further investigate chiasma formation in *dot-1*.*1* mutants, we scored the number of DAPI-stained bodies observed in -1 oocytes at diakinesis (the most proximal oocyte to the spermatheca) in wild-type, *ced-3* and *dot-1*.*1; ced-3* worms (Fig 5B). While 100% of -1 oocytes in wild-type and *ced-3* mutant worms contained 6 DAPI-stained bodies (bivalents), consistent with 6 pairs of attached homologs (n = 38 and 48, respectively), only 87.4% (n = 48) of oocytes in *dot-1*.*1; ced-3* worms exhibited 6 DAPI-stained bodies with 6.3% each carrying 5 and 7 DAPI-stained bodies, respectively (3/48 oocytes each). The presence of 5 DAPI-stained bodies suggests potential end-to-end chromosome fusions or aggregates, which might be due to the telomere effects of DOT-1 [43] or could reflect ectopic recombination events. 7 DAPI-stained bodies suggest the presence of 5 bivalents and two univalents, which might be due to problems with CO homeostasis in the *dot-1*.*1* mutant. Careful examination of chromosome morphology revealed significantly elevated levels of -1 oocytes with aberrant chromosome condensation in *dot-1*.*1*; *ced-3* worms (25% compared with 0% in wild type and 6.25% in *ced-3* single mutants, P<0.0001; Fisher’s exact test). Furthermore, albeit not statistically significant, *dot-1*.*1* mutants exhibited additional chromosome morphology defects including presence of fragments (4.16% compared to 0% in wild type and *ced-3* single mutant), frayed appearance (6.25% compared to 2.6% in wild type and 4.16% in *ced-3* single mutant), and aggregates (6.25% compared to 0% in wild type and *ced-3* single mutant) (Fig 5C). To analyze chromosome condensation more quantitatively, we measured the area occupied by each DAPI-stained body from -1 oocytes analyzed in wild-type, *dot-1*.*1; ced-3* and *ced-3* mutant worms. In wild-type worms, -1 oocytes have 6 well-condensed DAPI-stained bodies, so decondensation would result in an increase in the area occupied by each pair of attached homologs. The mean area per DAPI-stained body was 1.31 μm^2^ and 1.32 μm^2^ for wild-type (n = 228) and *ced-3* mutant worms (n = 288), respectively. The mean area per DAPI-stained body was significantly increased for *dot-1*.*1; ced-3* mutants, reaching 1.80 μm^2^ (n = 288, P<0.0001; by the two-tailed Mann-Whitney test, 95% C.I.; Fig 5D). Taken together, these data support a role for DOT-1.1 in promoting normal CO levels and maintenance of genomic integrity.

### DOT-1.1 regulates a meiotic checkpoint in worms

**Fig 6:**
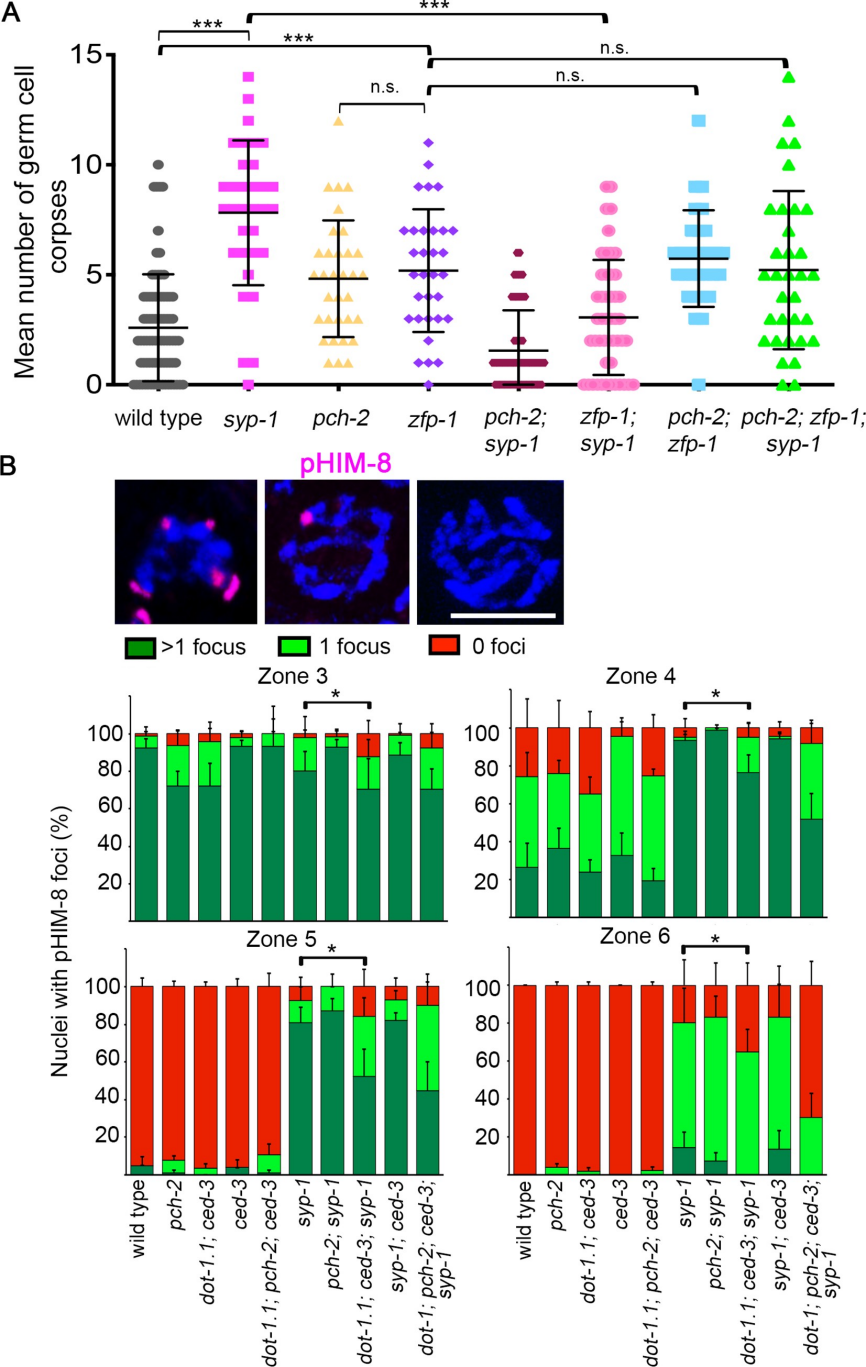
H3K79me is required for the synapsis checkpoint. **(A)** Scatter plot showing the distribution of the numbers of germ cell corpses detected in hermaphrodites from the indicated genotypes. Bars indicate mean ± SD. Levels of germ cell corpses in *syp-1* mutant worms were significantly higher than those observed in wild-type worms. However, such increase is no longer observed in a *zfp-1; syp-1* double mutant (***P<0.0001 by the two-tailed Mann-Whitney test, 95% C.I., after Bonferroni correction). A minimum of 27 gonads per genotype were scored from two to three biological repeats. n.s. = not significant, by the two-tailed Mann-Whitney test, 95% C.I. **(B)** Top, high magnification images of representative nuclei showing the different categories of phospho-HIM-8 (pHIM8) foci scored (the nucleus with >1 focus corresponds to zone 3, 1 focus corresponds to zone 4 and 0 foci corresponds to zone 5). Scale bar, 5 μm. Bottom, histogram representing the percentage of nuclei with >1, 1 or 0 pHIM-8 foci. In wild-type worms most of the nuclei show more than 1 focus in zone 3, even distribution of >1 and 1 focus in zone 4 and mostly 0 foci reaching zone 5. *syp-1* mutant worms never show 100% of germline nuclei with 0 foci; however, germline nuclei in *dot-1*.*1; ced-3; syp-1* show an increase in the percentage of nuclei exhibiting 0 foci in mid- to late pachytene (zones 5 and 6). The zone quantified is indicated on top of each graph. 4 to 6 gonads from two biological repeats were analyzed. Error bars represent SEM. *P<0.02 by the Chi-square test, after Bonferroni correction.

To explore the possibility that H3K79me is required for a meiotic checkpoint in worms, as observed in *S*. *cerevisiae* [18,21], we examined germ cell apoptosis levels. However, since the *dot-1*.*1* mutant used in this study must be combined with a *ced-3* mutation to maintain viability, we were unable to score germ cell apoptosis in this background lacking a caspase, and instead examined apoptosis in a *zfp-1* mutant. The *zfp-1* gene is the worm homolog of the MLL fusion partner, acute lymphoblastic leukemia 1-fused gene from chromosome 10 (AF10), and ZFP-1 has been shown to interact directly with DOT-1.1 modulating its histone methyltransferase activity [19]. Moreover, ZFP-1::GFP expression in the gonads of adult worms, with most prominent localization to oocyte chromosomes, has been previously described [44]. *zfp-1* encodes for two predicted protein isoforms (long and short; [44]). The *zfp-1(gk960739)* mutant carries a deletion that removes the first 109 amino acids from the long isoform of ZFP-1 as shown by Western blot analysis with a C terminus-specific ZFP-1 antibody [44] (S2 Fig), and is not predicted to be a null mutant. In agreement with this, while we observed a significant decrease in H3K79 mono-, di- and trimethylation signal in the gonads of *zfp-1* mutant worms compared to wild type, H3K79me levels were not as decreased as in the *dot-1*.*1; ced-3* null mutant (Fig 1). Importantly, analysis of meiotic progression, homolog pairing, chromosome synapsis, RAD-51 foci levels and chromosome morphology defects at diakinesis in *zfp-1* mutants revealed similar meiotic defects as those observed in *dot-1*.*1* mutants supporting the use of the *zfp-1* mutant as a proxy for *dot-1*.*1* in the analysis of germ cell apoptosis (S3 and S4 Figs and S1 Appendix). In order to trigger meiotic checkpoints in worms, we used the *syp-1* mutant that lacks SC formation and has been previously shown to exhibit both synapsis checkpoint- and DNA damage checkpoint-dependent elevated germ cell apoptosis [31]. As a control, we also analyzed the *pch-2* mutant implicated in the checkpoint that monitors chromosome synapsis in *C*. *elegans* [45]. Thus, we examined the levels of germ cell apoptosis by acridine orange staining in *syp-1*, *pch-2* and *zfp-1* single mutants as well as the combination of double and triple mutants. As expected, germ cell apoptosis was dramatically increased in *syp-1* compared to wild type (P< 0.0001 by the two-tailed Mann-Whitney test, 95% C.I.) (Fig 6A) and, as previously described, this enhanced apoptosis was reduced in the synapsis checkpoint-defective *syp-1; pch-2* double mutant (P< 0.0001, by the two-tailed Mann-Whitney test, C.I. 95%) [45]. Interestingly, like *pch-2; syp-1*, the *zfp-1; syp-1* double mutant also displayed significantly decreased levels of apoptotic corpses compared to *syp-1* (P< 0.0001, by the two-tailed Mann-Whitney test, 95% C.I.) (Fig 6A), suggesting that the reduced H3K79me observed in the absence of ZFP-1 leads to impaired meiotic checkpoint function. Although apoptotic levels were increased in the *zfp-1* single mutant relative to wild type, they were similar to those in *pch-2*, *pch-2*; *zfp-1;* and *pch-2; zfp-1; syp-1* mutants (Fig 6A, S1 and S2 Tables), suggesting that the small increase in germ cell apoptosis observed in the *zfp-1* single mutant does not result from activation of the PCH-2-dependent checkpoint. Thus, these results suggest that regulation of H3K79me levels is important for the surveillance mechanism that monitors proper synapsis in *C*. *elegans*.

Additionally, quantification of the levels of RAD-51 foci revealed that, like *pch-2*, mutation of *dot-1*.*1* also alters the number of RAD-51 foci in a *syp-1* mutant background (Fig 4B). In a *syp-1* mutant, the number of RAD-51 foci is drastically increased and persists for longer due to an inability to repair DSBs from a homologous partner since homologs are not stably held together in the absence of the SC [37]. However, in the *dot-1*.*1; ced-3; syp-1* triple mutant, the mean number of RAD-51 foci decreased significantly starting from transition zone (zone 3; Fig 4B), suggesting a disruption in the activation of the synapsis checkpoint. The reduction in the levels of RAD-51 foci in the absence of DOT-1.1 comes probably from a reduction in the total number of DSBs generated (Fig 4D), although we cannot rule out the possibility that some of the observed decrease is due to an ability to repair a fraction of DSBs.

As another approach to investigate the possible role of H3K79me in the synapsis checkpoint, we monitored CHK-2 activity in *syp-1* and *dot-1*.*1; syp-1* mutants. In yeast, it is known that Dot1 affects activity of Mek1 (the CHK-2 ortholog) [18], so we explored whether this mechanism is conserved in worms. As a proxy for CHK-2 activity we used the phosphorylation status of HIM-8, which is CHK-2-dependent [46]. We measured CHK-2 activity by quantifying the fraction of nuclei with 1 focus, >1 foci or 0 foci for phosphorylated HIM-8 (pHIM-8) in leptotene/zygotene (zone 3) and pachytene stages of meiosis (zones 4 to 6) (Fig 6B). The distribution of pHIM-8 in the germline of *dot-1*.*1* mutants was similar to wild type, with predominantly 0 foci observed by mid-pachytene when homologs are fully paired and synapsed and pHIM-8 is no longer observed (Fig 6B). As previously described, we observed that CHK-2 activity was prolonged in the *syp-1* mutant in response to synapsis failure [46], and we found that this striking extension was significantly reduced in *dot-1*.*1; syp-1* double mutants, supporting a possible role for DOT-1-dependent H3K79 methylation in the *C*. *elegans* meiotic checkpoint sensing chromosome synapsis (zones 3–6, P<0.02, Chi-square test) (Fig 6B).

**Fig 7:**
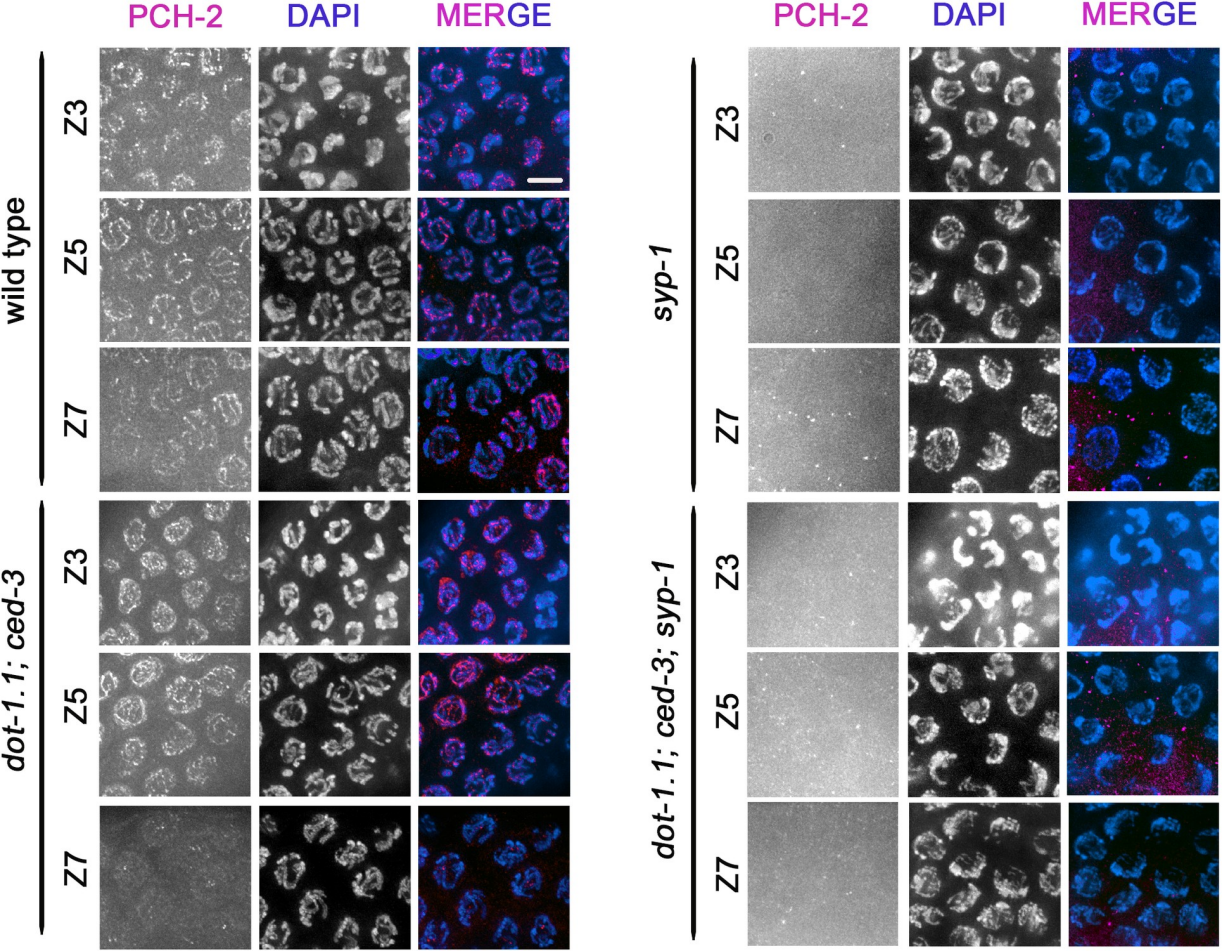
H3K79me does not regulate PCH-2 localization in *C*. *elegans*. High-resolution images of germline nuclei from the indicated genotypes co-stained with PCH-2 (magenta) and DAPI (blue). In wild-type and *dot-1*.*1; ced-3* worms PCH-2 starts to form tracks on chromosomes at transition zone (Z3). PCH-2 localizes to the SC until mid-pachytene (zone 5) and its signal diminishes by late pachytene as the SC disassembles (Z7). PCH-2 does not localize to chromosomes and instead is detected as a diffuse non-chromatin associated signal in *syp-1* and *dot-1*.*1; ced-3; syp-1* mutants. Scale bar, 5 μm.

Finally, in yeast, it has been proposed that Dot1 modulates the meiotic checkpoint response in part by regulating Pch2 localization. In the yeast *zip1Δ dot1Δ* double mutant, the nucleolar confinement of Pch2 is lost correlating with defective checkpoint response [18]. To analyze whether *C*. *elegans* uses a similar mechanism we evaluated PCH-2 localization in *dot-1*.*1*, *syp-1* and *dot-1*.*1; syp-1* mutants. As previously shown, PCH-2 is present in germline nuclei prior to the transition zone, it localizes to the SC upon entrance into meiosis, and is diminished by late pachytene [47] (Fig 7). In *dot-1*.*1; ced-3* worms the distribution of PCH-2 is indistinguishable from wild type (Fig 7), which suggests that H3K79me does not regulate PCH-2 localization to the SC under normal conditions. Like in yeast, synapsis is required for PCH-2 localization to the SC, since PCH-2 localization is completely lost from chromosomes in *syp-1* mutants and is observed only as a diffuse background-like signal [47] (Fig 7). However, unlike yeast, PCH-2 localization to the rDNA is not observed in *C*. *elegans* [47] and analysis of PCH-2 in the *dot-1*.*1; syp-1* mutant did not show alteration of the diffuse PCH-2 distribution. Thus, *pch-2* and *dot-1*.*1* may be working in different pathways to promote a chromosome synapsis checkpoint in *C*. *elegans*. Consistent with this notion, a *dot-1*.*1; pch-2; ced-3; syp-1* quadruple mutant exhibited a decrease in the number of nuclei with aberrant CHK-2 activity compared to that of *dot-1; ced-3; syp-1* and *pch-2; syp-1* double mutants (zone 6, P<0.003 and P<0.0003 respectively, Chi-square test) (Fig 6B).

## Discussion

### DOT-1.1’s roles in promoting embryonic viability and accurate chromosome segregation

The decreased brood size and increased embryonic lethality observed in the *dot-1*.*1* mutant can be due in part to defects during meiosis leading to errors in chromosome segregation and the consequent formation of aneuploid gametes, as has been previously shown for meiotic mutants in *C*. *elegans* [48,49]. Besides problems with chromosome segregation, embryonic lethality can result from problems in early embryo development. We cannot discard the possibility that the embryonic lethality observed in *dot-1*.*1* mutants is the consequence of early developmental problems as it has been demonstrated in mice and flies. Specifically, germline knockout of *mDOT1L* results in lethality by embryonic day 10.5 (E10.5) during organogenesis of the cardiovascular system [22]. Furthermore, Grappa, the homolog of DOT1L in *Drosophila*, plays an important role in regulating transcription of developmental genes [25]. In general, DOT1L has been implicated in regulating gene expression due to its activity as a methyltransferase. Nevertheless, although Dot1/DOT1L-dependent H3K79me preferentially occurs at actively transcribed ORFs, there are only few cases where Dot1/DOT1L has been causally linked to transcription regulation [50]. Studies in *C*. *elegans* suggest that DOT-1.1/H3K79 methylation at the promoters of ubiquitously expressed genes may promote RNA polymerase II pausing [19]. Here, we showed that H3K79me levels decrease in the germline of *dot-1*.*1* mutant worms, so it is likely that DOT-1.1 is regulating gene expression levels in the germline. Although DOT1L is the only H3K79 methyltransferase in mammals and H3K79 methylation is present on actively transcribed genes, inhibition of DOT1L methyltransferase activity does not result in global dramatic changes in gene expression in cultured cells [51]. However, expression of specific genes, such as *HOXA9* and *MEIS1*, is strongly dependent on DOT1L specially in leukemias induced by MLL-fusion proteins [52]. In these cases, DOT1L promotes gene expression through antagonizing local heterochromatin [53,54]. Therefore, embryonic lethality and reduction in brood size may be related to the direct regulation of the chromatin environment at specific gene loci. Moreover, particular defects observed with chromosome morphology in oocytes at diakinesis suggest specific gene expression regulation by DOT-1.1. Notably, problems at the level of chromosome compaction (Fig 5) can result from the direct regulation of genes such as *arf-1*.2 (ortholog of human ARF1, ADP ribosylation factor 1) and *rnr-2* (ortholog of human RRM2, ribonucleotide reductase regulatory subunit M2) which have been described as potential targets of DOT-1.1 and implicated in oocyte chromatin condensation [19,55]. Both the *arf-1*.*2* and *rnr-2* loci contain extended domains of DOT-1.1 binding, which is a notable feature of mammalian *HOXA9* and *MEIS1* [53,54], as well as of the lineage-specific *C*. *elegans* genes positively regulated by DOT-1.1 [23]. Thus, the reduction of H3K79me increased the occurrence of chromosomal abnormalities, which is consistent with the increase in sterility and embryonic lethality, revealing significant defects in genomic stability.

### Decreased DSB formation and altered CO designation levels in *dot-1*.*1* suggest alterations in chromosome structure

Our experiments showed a reduction in the levels of DSB formation and deregulation of CO formation (Fig 4 and Fig 5) in the *dot-1*.*1* mutant worms, which may be directly related to the observed decrease in the levels of H3K79me in this mutant (Fig 1). Numerous enzymes have been shown to catalyze post-translational modifications of core histone proteins, and each of these modifications has profound impacts on overall chromatin organization [56,57]. Moreover, the organization of large-scale chromatin architecture in prophase I meiocytes has been attributed a role in the global modulation of meiotic recombination and CO frequency [58–60]. One key piece of evidence to substantiate this model is that the frequency of MLH1 foci, a CO marker, is more closely associated with the length of the SC than with DSB frequency [61]. Therefore, the relation between chromatin modifications and the lengths of the chromosome axes as well as of the chromatin loops is essential for the establishment of chromosome structure and regulation of gene expression. In *C*. *elegans*, elongation of chromosome axes in condensin mutants showed that perturbations to chromosome structure influence the position and frequency of DSBs in the genome and, hence, of COs [39]. Thus, a potential explanation for CO deregulation in the *dot-1*.*1* mutant is the alteration of the chromatin landscape derived from the depletion of H3K79me with potential implications in the deregulation of either chromatin loops or chromosome axes. Indeed, yeast Dot1 competes with the heterochromatic Sir proteins for binding to the histone H4 tail thus preventing heterochromatin spreading from telomeres to central chromosome regions [62,63]. Moreover, yeast Dot1 also promotes meiotic DSB formation in the absence of Set1-dependent H3K4 methylation suggesting that the interplay between different chromatin modifications is important to establish the proper meiotic DSB landscape [64]. However, the overall structure of metazoan DOT1L proteins is distinct from the yeast one [19] and DOT1L relies on partners, such as AF10/ZFP-1 and AF9, for chromatin localization [52,65,66]. Nevertheless, DOT1L/H3K79me has been implicated in preventing the deposition of silencing chromatin marks [53,54], although the precise molecular mechanism of this is not yet clear. Thus, H3K79 methylation depletion in germline chromatin in *C*. *elegans* very likely affects global chromosome architecture. Another non-mutually exclusive possibility is the regulation of the expression of specific genes involved in either DSB formation and/or CO designation. DOT-1.1-dependent regulation of *spo-11* expression, the gene encoding for the topoisomerase-like factor that catalyzes meiotic DSBs [67], is not likely given that *dot-1*.*1* mutants do not exhibit the 12 univalents at diakinesis normally associated with the complete lack of DSB formation and subsequent CO formation. However, we cannot rule out the possibility that *dot-1*.*1* regulates the expression of other genes modulating DSB formation.

### H3K79me regulates a meiotic checkpoint in *C*. *elegans*

Proper chromosome segregation relies on the accurate interaction between homologous chromosomes, which includes synapsis and recombination. During meiosis in *C*. *elegans*, checkpoints are set in place to monitor pairing, synapsis and recombination. Here we showed evidence that a meiotic checkpoint surveilling synapsis is misregulated in *dot-1*.*1* mutants. We suggest that such misregulation is directly connected to the decrease in H3K79me levels observed in *dot-1*.*1* germline as has been shown for yeast, where the status of H3K79 methylation modulates the meiotic recombination checkpoint, with the H3K79me3 form being the most relevant to sustain the checkpoint response [18]. Unlike yeast, where the Dot1 protein is dispensable in otherwise unperturbed meiosis, we found that in *C*. *elegans* DOT-1.1 has a role in the regulation of key meiotic processes: pairing, synapsis and recombination. This is closer to the general effects observed for *dot1* mutants in evolutionarily higher organisms, suggesting that H3K79me function has evolved in metazoans. We show evidence that CHK-2 activity (measured by pHIM-8) is reduced in synapsis-defective mutants when they are in combination with a *dot-1*.*1* mutation. CHK-2 is essential for DSB formation and acts as a master regulator that governs pairing, synapsis, and recombination during meiotic prophase [68]. Thus, the reduced number of DSBs in *dot-1*.*1; ced-3; syp-1* worms may stem from impaired CHK-2 activity. Checkpoint regulation by DOT-1.1 seems to be independent of axis proteins since the HORMA domain protein HTP-3 is mostly not affected in *dot-1*.*1* mutants. However, it remains to be determined if DOT-1.1 directly regulates the expression/activity of *chk-2*.

The defects in chromosome synapsis and the generation of aneuploid gametes (Fig 2) are still manifested in the *dot-1*.*1; syp-1* double mutant despite the kinetics of meiotic progression being partially rescued in this background (Fig 6). Therefore, relief of the meiotic block by the *dot-1*.*1* mutation is likely not due to suppression of the defects that trigger checkpoint-induced arrest, but rather due to disruption of the checkpoint *per se* as has been proposed in yeast [18,21]. The mechanism by which H3K79me, a constitutive histone mark, is regulating the checkpoint activation needs to be clarified. Like in *syp-1* worms, in yeast *zip1Δ* mutants lacking the central region of the SC, Pch2 is lost from chromosomes. However, unlike worms, Pch2 remains associated to the unsynapsed nucleolar rDNA array in yeast *zip1Δ* [69,70]. In the checkpoint-defective yeast *zip1Δ dot1Δ* mutant, Pch2 is not retained in the nucleolus and instead it distributes throughout chromatin leading to the proposal that regulation of Pch2 nucleolar localization by Dot1 is important for checkpoint function [69,70]. However, more recent studies have demonstrated that the Pch2 protein also localizes in the cytoplasm of yeast cells, and that the presence of Pch2 in the nucleolus is actually dispensable for checkpoint function [71]. In *syp-1* worms, PCH-2 is not detected associated to chromatin [47] (Fig 7), but the synapsis checkpoint is active [45] (Fig 6A). All these observations raise the question, both in yeast and *C*. *elegans*, of where the Pch2 protein relevant for the checkpoint is localized. Thus, it is conceivable that the impact of DOT-1.1 in the *syp-1*-induced meiotic checkpoint may not be directly linked to PCH-2 chromosomal distribution. It is possible that DOT-1.1 is acting through a mechanism more similar to the one proposed in mammals for DNA damage checkpoint activation where chromatin remodeling in the vicinity of DNA lesions may locally expose histone marks (i.e., H3K79me, H4K20me) supporting the recruitment of DNA damage checkpoint adaptors to activate the checkpoint [72,73]. Therefore, when the histone mark is not present, the recruitment of proteins is not activated and the checkpoint activation is abrogated.

## Material and methods

### Genetics

*C*. *elegans* strains were cultured at 20°C under standard conditions as described in [74]. The N2 Bristol strain was used as the wild-type background. The following mutations and chromosome rearrangements were used: linkage group I (LG1), *dot-1*.*1[knu337-(pNU1092-KO loxP*::*hygR*::*loxP)]*, *rad-54(ok615)/ht2[bli-4(e937)let-*?*(q782)qls48]* (I,III); LGII, *pch-2(tm1458)*; LGIII, *zfp-1(gk960739)*; LGIV, *ced-3(n1286)*; LGV, *syp-1(me17)/nt1[unc-*?*n754]let-*?*gls50)(IV;V)*. The *zfp-1(gk960739)* mutant has been outcrossed at least eight times from the VC40040 strain generated by the Million Mutation Project [75]. All other mutants have been outcrossed at least six times. Full genotypes for combinatorial mutants used in this study are listed in S3 Table.

### Scoring embryonic lethality, sterility and males

Age-matched (24 hours post-L4 larval stage) individual hermaphrodites were placed on regular NGM plates and assessed for embryonic lethality, sterility and the percentage of males among their progeny. Worms were moved every 24 hours to new NGM plates for four consecutive days. The total number of fertilized eggs laid, hatched, the number of progeny that reached adulthood, and the frequencies of male progeny, were scored.

### Cytological analysis

Whole mount preparations of dissected gonads and immunostainings were performed as in [68] with some modifications. Briefly, gonads from 24h post-L4 hermaphrodites were dissected and fixed with 1% formaldehyde for 5 minutes, freeze-cracked and post-fixed in ice-cold 100% methanol for 1 minute followed by blocking with 1% BSA for 1 hour. Gonads for RAD-51 immunostaining were dissected and then freeze-cracked and fixed in 4% formaldehyde for 30 minutes. The following primary antibodies were used at the indicated dilutions: rabbit α-phospho HIM-8 (1:1000, [46]), rabbit α-PCH-2 (1:500, [47]), rabbit α-H3K79me1 (1:500, Abcam Ab2886), rabbit α-H3K79me2 (1:500, Abcam Ab3594), rabbit α-H3K79me3 (1:500, Abcam Ab2621), goat α-SYP-1 (1:3000, [76]), rabbit α-HIM-8 (1:500, Novus Biological (SDI)), guinea pig α-HTP-3 (1:500, [35]), rabbit anti-RAD-51 (1:10,000; Catalog #29480002; Novus Biologicals (SDI)), guinea pig α-ZHP-3 (1:500, [77]), guinea pig α-SUN-1 Ser8-pi (1:700, [30]) and mouse α-Ack (Cell Signaling Technology, 1:1000). The secondary antibodies, from Jackson ImmunoResearch Laboratories (West Grove, PA), were used at the following dilutions: α-rabbit Cy-3 (1:200), α-mouse Cy-3 (1:200), α-guinea pig Cy-5 (1:100), α-goat Alexa 488 (1:500), α-rabbit Alexa 488 (1:500), and α-guinea pig Alexa 488 (1:500). DAPI was used to counterstain DNA. Vectashield from Vector Laboratories (Burlingame, CA) was used as a mounting media and anti-fading agent.

Quantitative analysis of pHIM-8 foci consisted of scoring the number of foci observed per nucleus for nuclei in all seven zones composing the germline. Between 4 and 6 gonads were scored for each genotype (S1 Table).

To evaluate chromosome morphology in oocytes at diakinesis, whole worms were Carnoy’s fixed and then stained with DAPI as in [78]. Images were taken from the diakinesis oocyte more proximal to the spermatheca (-1 oocyte).

Imaging was performed using an IX-70 microscope (Olympus) with a cooled CCD camera (model CH350; Roper Scientific) controlled by the DeltaVision system (Applied Precision). Images were collected using a 100x objective with or without auxiliary magnification (1.5x) and Z-stacks were set at 0.2 μm thickness intervals. Image deconvolution was done using the SoftWoRX 3.3.6 program (Applied Precision) and processed with Fiji ImageJ [79,80].

### Fluorescence in situ hybridization

Probe for the 5S rDNA locus (center of chromosome V) was generated and labeled as in [67]. Quantification of homologous pairing was performed as in [37], using germlines from age-matched worms 18–22 h post-L4 larval stage. Distances between peak intensities of FISH signals were measured and considered paired if ≤ 0.75 μm apart. Briefly, gonads were dissected in 1X egg buffer and fixed with 3.7% formaldehyde for 2 minutes. After freeze cracking, slides were incubated in ice-cold methanol for 30 minutes then washed 2 times with 2xSSC. Slides were incubated in 2xSSC containing 50% formamide for 2 hours prior to adding the FISH probe at 37°C. DNA was denatured for 90 sec at 93°C. Hybridization was carried out overnight at 37°C in a water bath. After hybridization, slides were washed 2 times with 2xSSC containing 50% formamide for 30 min at 37°C, 1 time with 2xSSCT containing 25% formamide at room temperature for 15 min, and 2 times with 2xSCCT for 10 min. DNA was counterstained with DAPI (Thermo Fisher Scientific D1306), destained with 2xSSCT for 1 hour, and mounted with VectaShield (Vector Laboratories H-1000). The average number of nuclei scored per zone (n) for wild type, *dot-1*.*1 ced-3*, *ced-3* and *zfp-1* are as follows: zone 1 (n = 84), zone 2 (n = 80), zone 3 (n = 85), zone 4 (n = 93), zone 5 (n = 95), zone 6 (n = 76), and zone 7 (n = 93). Statistical comparisons were performed using the Fisher’s exact test.

### Time course analysis for RAD-51 foci

Quantitative analysis of RAD-51 foci for nuclei in all seven zones composing the germline was performed as in [37]. The average number of nuclei scored per zone (n) from 4 to 6 gonads for each genotype ± standard deviation is shown in S1 Table. Statistical comparisons were performed using the two-tailed Mann-Whitney test, 95% C.I. (S2 Table).

### Germ cell apoptosis

Acridine orange (AO) staining of apoptotic germ cells in wild type (N2), *zfp-1*, *syp-1 and pch-2* mutants as well as in the corresponding double and triple mutants was performed as in [81]. Briefly, apoptotic germ cell corpses were scored in the germlines of 24 hours post-L4 hermaphrodites following incubation with AO for 2 hours at room temperature. The germlines of between 27 and 80 worms from at least two independent biological repeats were scored for each genotype. Apoptotic germ cell corpses were visualized using a Leica DM5000B fluorescence microscope. Statistical comparisons between genotypes were done using the two-tailed Mann-Whitney test, 95% C.I. (S2 Table).

### Western blot analysis

Adult worm lysates were resolved via SDS-PAGE on a 4 to 12% gel (Invitrogen NuPAGE Bis-Tris gel) and transferred to a 4.5-μm nitrocellulose membrane (Bio-Rad) at 100 mA for 1 h. The membrane was blocked using 3% (wt/vol) BSA in PBS with 0.01% Tween for 1 h, followed by overnight incubation at 4°C with an anti-ZFP-1 C-terminus-specific antibody [82], diluted 1:2,000 in PBST-3% BSA. The membrane was then washed 3 times with PBST, incubated for 1 h at room temperature with a horseradish peroxidase (HRP)-conjugated anti-rabbit secondary antibody (PerkinElmer) diluted 1:5,000 in PBST-3% BSA, and visualized by using SuperSignal West Pico chemiluminescence substrate (Thermo Scientific) and a series 2000A film processor (Tiba).

## Supporting information

S1 Fig H3K79me signal is evenly distributed on chromosomes from pachytene nuclei in wild-type germlines. High-resolution images of early pachytene nuclei (zone 4) from wild-type germlines co-stained with H3K79me1 (upper panel, green), H3K79me2 (middle panel, green) or H3K79me3 (lower panel, green), AcK (magenta) and DAPI (blue). In wild-type gonads, AcK is enriched on autosomes compared to the X chromosomes, while H3K79me1,-2,-3 signals are detected on all chromosomes. Yellow arrowheads indicate X-chromosome based on nearly absent AcK signal. Scale bar, 5 μm.
(TIF)

S2 Fig Western blot analysis showing whole worm lysates from wild-type and *zfp-1(gk960739)* adult worms immunoblotted with an anti-ZFP-1 C-terminus-specific antibody. The top band represents the long isoform (above 100 kDa) and the lower band corresponds to the short isoform (predicted at 65 kDa). The deletion in *zfp-1(gk960739)* removes the first 109 amino acids from the long isoform.
(TIF)

S3 Fig *zfp-1* mutant worms exhibit defects in meiotic progression, delayed pairing and problems with SYP-1 loading. **(A)** Whole mounted gonads of wild-type and *zfp-1* worms co-immunostained with SUN-1(pS8) (green) and DAPI (blue). *zfp-1* mutant worms showed an extension in SUN-1-positive signal compared to wild type suggesting defects in meiotic progression. Asterisks indicate the premeiotic tip and white arrows show the direction in which nuclei move proximally in the germline (meiotic progression). Hatched lines demarcate the first (left) and the last (right) rows with nuclei showing SUN-1 (pS8) signal. At least 30 gonads from two independent biological repeats were analyzed for each genotype. Scale bars, 5 μm. **(B)** Top left, high-resolution images of early pachytene nuclei (zone 4) co-stained with HIM-8 (magenta) and DAPI (blue). Yellow arrow indicates nucleus with unpaired HIM-8 signal. Scale bar 5 μm. Bottom left, histogram representing the percentage of nuclei with paired HIM-8 signals scored at different zones along the germline in wild-type and *zfp-1* worms. X chromosomes were scored as paired when the two HIM-8 signals were ≤ 0.75 μm apart from each other. * P<0.05, *** P<0.001, Fisher’s exact test. Top right, high-resolution images of early pachytene nuclei (zone 4) stained with DAPI and hybridized with a FISH probe against the 5S rDNA locus located near the center of chromosome V (green). Yellow arrow indicates nucleus with unpaired FISH signals. Scale bar 5 μm. Right bottom, histogram representing the percentage of nuclei with paired FISH signal (5S rDNA) scored at different zones along the germline in wild type and *zfp-1*. Chromosomes were scored as paired when two signals were ≤ 0.75 μm apart from each other. *P<0.025, ***P<0.0005, Fisher’s exact test. **(C)** Left, histogram indicating the percentage of nuclei that exhibit complete synapsis as a function of meiotic progression in wild-type and *zfp-1* gonads. Nuclei showing complete overlapping signal of the lateral element component HTP-3 and the central region component SYP-1 along all chromosomes were considered as nuclei with complete synapsis. 4 gonads from two biological repeats were scored for wild type (n = 154) and *zfp-1* (n = 173); n = number of nuclei scored. ***P<0.001, Fisher’s exact test. TZ, transition zone; EP, early pachytene; MP, mid-pachytene; LP, late pachytene. Right, high-resolution images of mid-pachytene nuclei co-immunostained with SYP-1 (green), HTP-3 (red), HIM-8 (yellow) and DAPI (blue). Unlike wild-type worms, 5.78% of nuclei (10/173) in *zfp-1* mutants exhibited DAPI-stained regions lacking SYP-1 signal at the pachytene stage (arrow). 10% (1/10) of these nuclei exhibited discontinuities in HTP-3 signal and in 70% (7/10) the unsynapsed chromosome corresponded to the X chromosome as determined by presence of HIM-8 signal (arrowhead). Scale bar, 5 μm.
(TIF)

S4 Fig *zfp-1* mutant worms show altered DSB formation and bivalent morphology defects. **(A)** High-resolution images representative of mid-pachytene nuclei (zone 5) immunostained for RAD-51 (magenta) and co-stained with DAPI (blue). Scale bar, 5 μm. **(B)** Histogram shows the mean number of RAD-51 foci/nucleus (y-axis) scored along each zone in the germlines (x-axis) of the indicated genotypes. Between 4 and 6 gonads were scored per genotype. A significant decrease in levels of RAD-51 foci were observed for zones 3 to 5 in *zfp-1* germlines compared to wild type and for zones 3 to 7 in *zfp-1*; *syp-1* germlines compared to *syp-1*. Error bars represent SEM for technical repeats from two biological replicates. ***P<0.0003 by the two-tailed Mann-Whitney test, 95% C.I., after Bonferroni correction. TZ, transition zone; EP, early pachytene; MP, mid-pachytene; LP, late pachytene. **(C)** Table shows the percentage of -1 oocytes at diakinesis displaying each one of the indicated defects in chromosome morphology. n = number of -1 oocytes scored. Decond. = decondensation. Aggreg. = aggregates.
(TIF)

S1 Table Raw data sets. Raw data for plate phenotyping, RAD-51 foci counts in wild-type and *rad-54* backgrounds, germ cell apoptosis counts and pHIM-8 foci counts.
(XLSX)

S2 Table Statistical analyses. P-values for comparisons among all genotypes used in this study using the two-tailed Mann-Whitney test, 95% C.I. P-values for RAD-51 foci counts in wild-type and *rad-54* backgrounds, and germ cell apoptosis.
(XLSX)

S3 Table Strains. List of strains used in this study.
(DOCX)

S1 Appendix *zfp-1* mutant worms exhibit similar phenotypes to those observed in *dot-1*.*1* worms. (DOCX)
