# Supplementary figures and images for: DOT-1.1-dependent H3K79 methylation promotes normal meiotic progression and meiotic checkpoint function in C. elegans
Source: PLoS Genet. 2020 Oct 26;16(10):e1009171. doi: 10.1371/journal.pgen.1009171 (PMC7644094; doi:10.1371/journal.pgen.1009171)

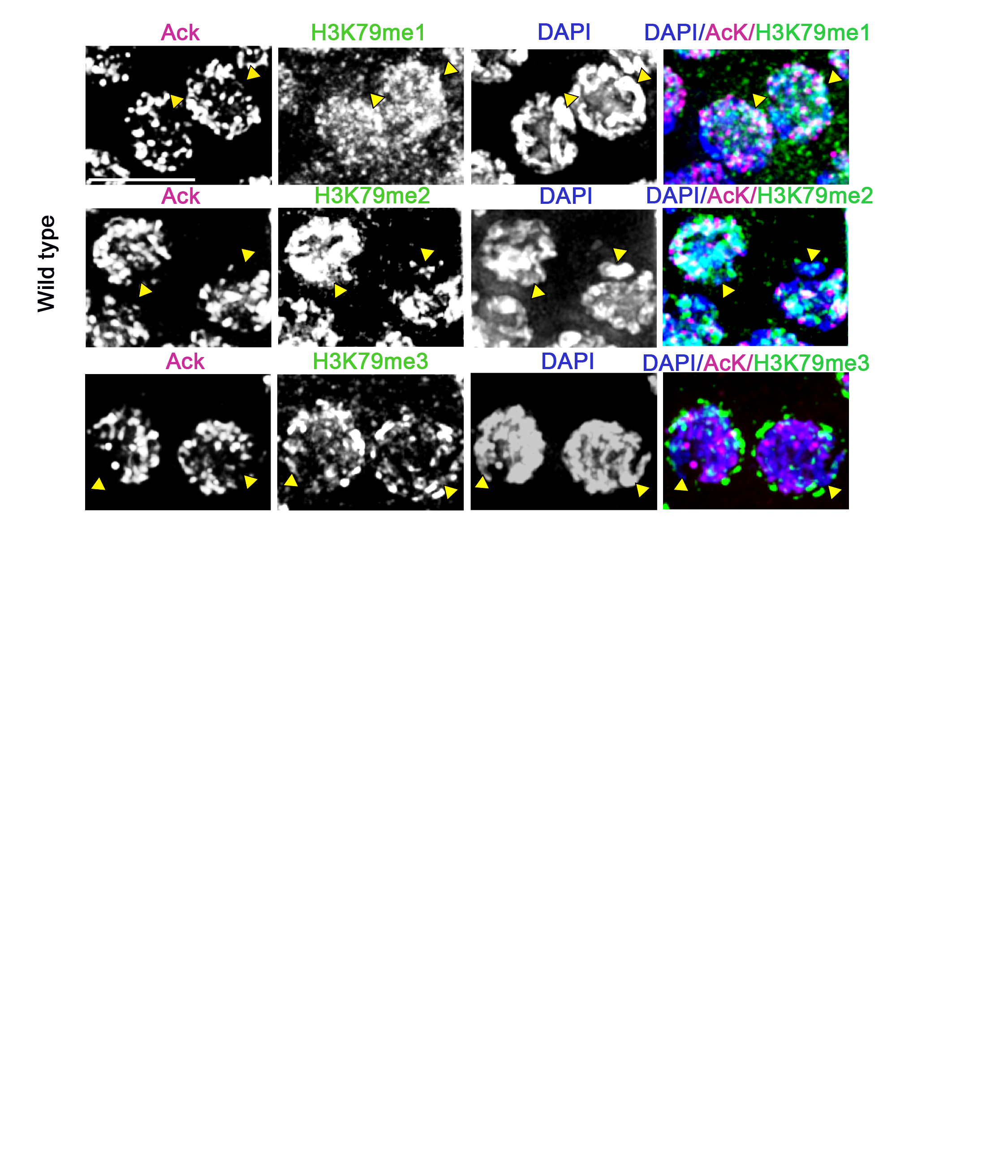

Supplement: S1 Fig — High-resolution images of early pachytene nuclei (zone 4) from wild-type germlines co-stained with H3K79me1 (upper panel, green), H3K79me2 (middle panel, green) or H3K79me3 (lower panel, green), AcK (magenta) and DAPI (blue). In wild-type gonads, AcK is enriched on autosomes compared to the X chromosomes, while H3K79me1,-2,-3 signals are detected on all chromosomes. Yellow arrowheads indicate X-chromosome based on nearly absent AcK signal. Scale bar, 5 μm. (TIF) [file pgen.1009171.s001.tif]

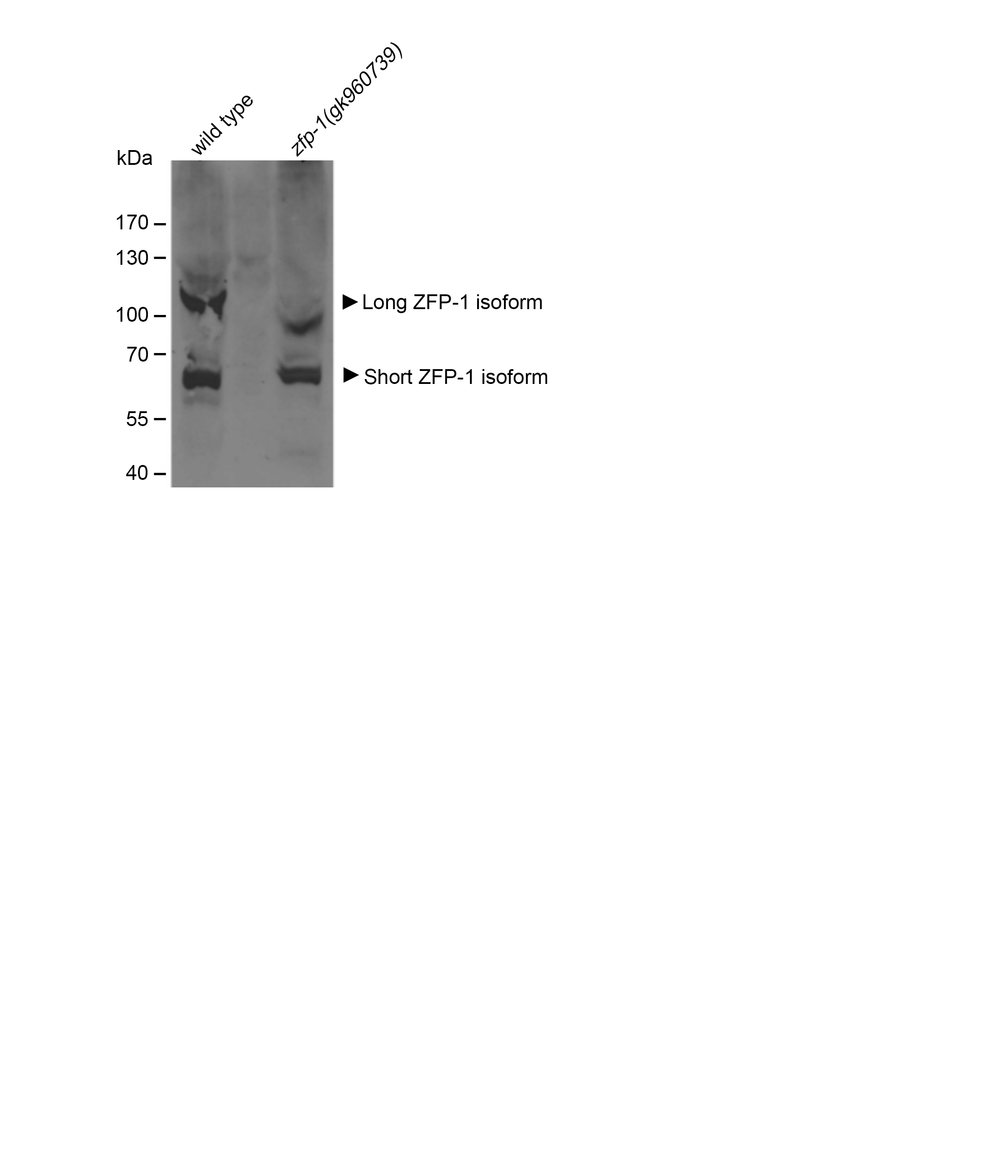

Supplement: S2 Fig — The top band represents the long isoform (above 100 kDa) and the lower band corresponds to the short isoform (predicted at 65 kDa). The deletion in zfp-1(gk960739) removes the first 109 amino acids from the long isoform. (TIF) [file pgen.1009171.s002.tif]

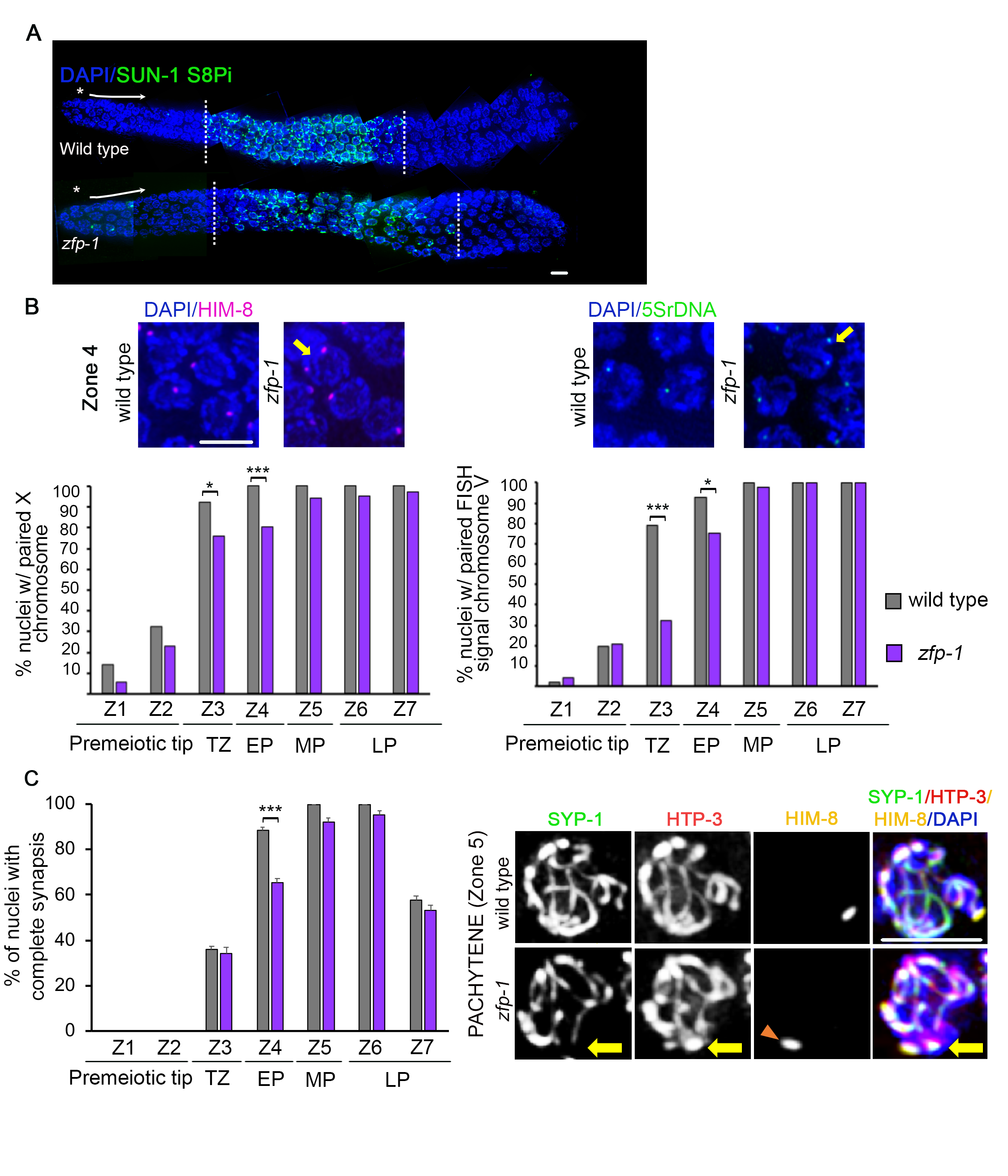

Supplement: S3 Fig — (A) Whole mounted gonads of wild-type and zfp-1 worms co-immunostained with SUN-1(pS8) (green) and DAPI (blue). zfp-1 mutant worms showed an extension in SUN-1-positive signal compared to wild type suggesting defects in meiotic progression. Asterisks indicate the premeiotic tip and white arrows show the direction in which nuclei move proximally in the germline (meiotic progression). Hatched lines demarcate the first (left) and the last (right) rows with nuclei showing SUN-1 (pS8) signal. At least 30 gonads from two independent biological repeats were analyzed for each genotype. Scale bars, 5 μm. (B) Top left, high-resolution images of early pachytene nuclei (zone 4) co-stained with HIM-8 (magenta) and DAPI (blue). Yellow arrow indicates nucleus with unpaired HIM-8 signal. Scale bar 5 μm. Bottom left, histogram representing the percentage of nuclei with paired HIM-8 signals scored at different zones along the germline in wild-type and zfp-1 worms. X chromosomes were scored as paired when the two HIM-8 signals were ≤ 0.75 μm apart from each other. * P<0.05, *** P<0.001, Fisher’s exact test. Top right, high-resolution images of early pachytene nuclei (zone 4) stained with DAPI and hybridized with a FISH probe against the 5S rDNA locus located near the center of chromosome V (green). Yellow arrow indicates nucleus with unpaired FISH signals. Scale bar 5 μm. Right bottom, histogram representing the percentage of nuclei with paired FISH signal (5S rDNA) scored at different zones along the germline in wild type and zfp-1. Chromosomes were scored as paired when two signals were ≤ 0.75 μm apart from each other. *P<0.025, ***P<0.0005, Fisher’s exact test. (C) Left, histogram indicating the percentage of nuclei that exhibit complete synapsis as a function of meiotic progression in wild-type and zfp-1 gonads. Nuclei showing complete overlapping signal of the lateral element component HTP-3 and the central region component SYP-1 along all chromosomes were considered as nuclei wi [file pgen.1009171.s003.tif]

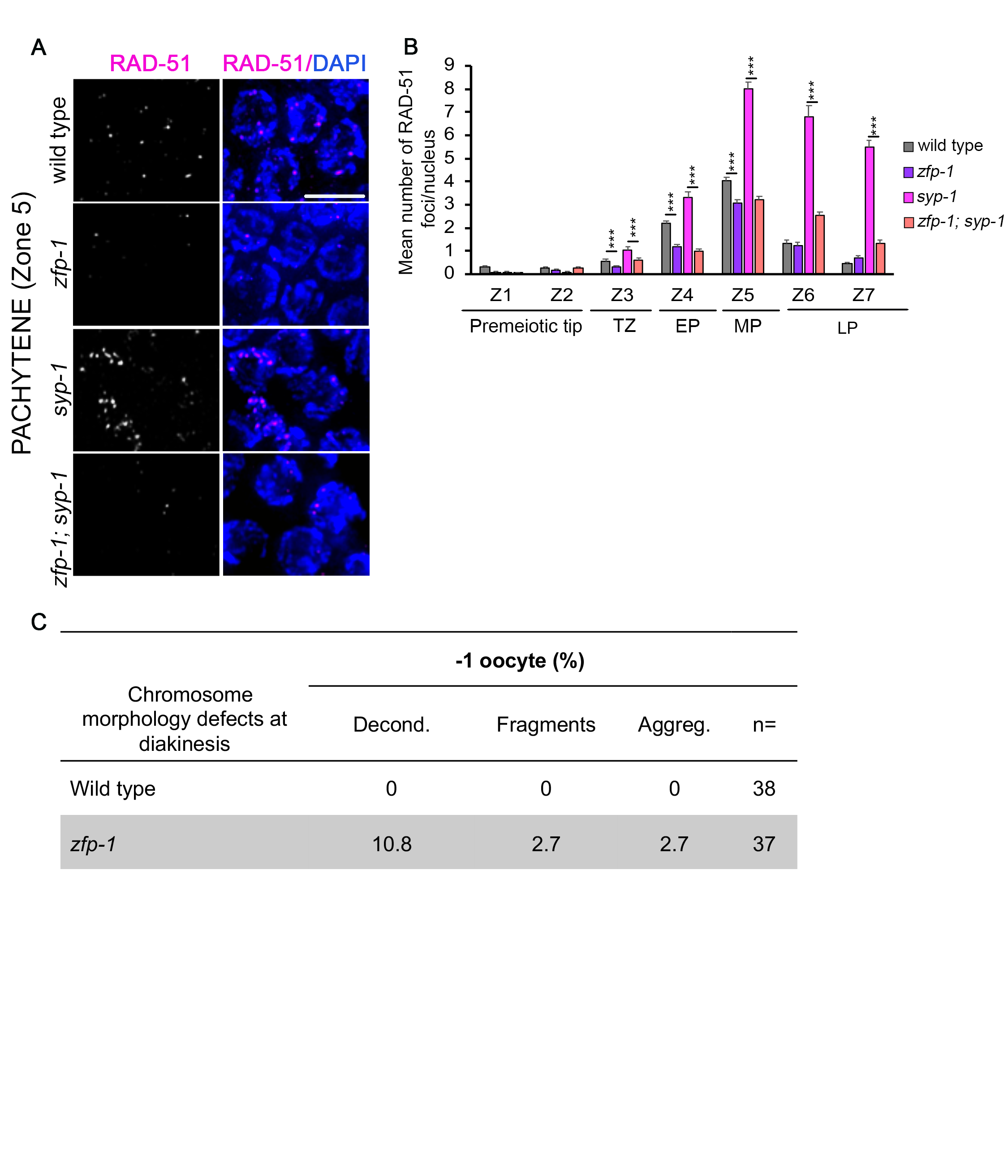

Supplement: S4 Fig — (A) High-resolution images representative of mid-pachytene nuclei (zone 5) immunostained for RAD-51 (magenta) and co-stained with DAPI (blue). Scale bar, 5 μm. (B) Histogram shows the mean number of RAD-51 foci/nucleus (y-axis) scored along each zone in the germlines (x-axis) of the indicated genotypes. Between 4 and 6 gonads were scored per genotype. A significant decrease in levels of RAD-51 foci were observed for zones 3 to 5 in zfp-1 germlines compared to wild type and for zones 3 to 7 in zfp-1; syp-1 germlines compared to syp-1. Error bars represent SEM for technical repeats from two biological replicates. ***P<0.0003 by the two-tailed Mann-Whitney test, 95% C.I., after Bonferroni correction. TZ, transition zone; EP, early pachytene; MP, mid-pachytene; LP, late pachytene. (C) Table shows the percentage of -1 oocytes at diakinesis displaying each one of the indicated defects in chromosome morphology. n = number of -1 oocytes scored. Decond. = decondensation. Aggreg. = aggregates. (TIF) [file pgen.1009171.s004.tif]
