# Supplementary material for: DOT-1.1-dependent H3K79 methylation promotes normal meiotic progression and meiotic checkpoint function in C. elegans
Source: PLoS Genet. 2020 Oct 26;16(10):e1009171. doi: 10.1371/journal.pgen.1009171 (PMC7644094; doi:10.1371/journal.pgen.1009171)
Supplement: S3 Table — List of strains used in this study. (DOCX) [file pgen.1009171.s007.docx]

**Table S3. *C. elegans* strains**

| **Strain Name** | **Genotype** |
| --- | --- |
| AGK769 | *zfp-1(gk960739)III* |
| AV176 | *syp-1(me17)V/nt1[unc-?n754]let-?gls50)(IV;V)* |
| COP1302 | *dot-1.1[knu337-(pNU1092-KO loxP::hygR::loxP)]I;ced-3(n1286)IV* |
| CV345 | *pch-2(tm1458)II* |
| CV592 | *pch-2(tm1458)II; syp-1(me17)V/nt1[unc-?n754]let-?gls50)(IV;V)* |
| CV775 | *pch-2(tm1458)II; zfp-1(gk960739)III* |
| CV776 | *zfp-1(gk960739)III*; *syp-1(me17)V/nt1[unc-?n754]let-?gls50)(IV;V)* |
| CV777 | *pch-2(tm1458)II;zfp-1(gk960739)III; syp-1(me17)V/nt1[unc-?n754]let-?gls50)(IV;V)* |
| CV816 | *dot-1.1[knu337-(pNU1092-KO loxP::hygR::loxP)]I; pch-2(tm1458)II; ced-3(n1286)IV; syp-1(me17)V/nt1[unc-?n754]let-?gls50)(IV;V)* |
| CV810 | *dot-1.1[knu337-(pNU1092-KO loxP::hygR::loxP)]I; pch-2(tm1458)II; ced-3(n1286)IV* |
| CV811 | *dot-1.1[knu337-(pNU1092-KO loxP::hygR::loxP)]I;ced-3(n1286)IV; syp-1(me17)V/nt1[unc-?n754]let-?gls50)(IV;V)* |
| CV824 | *ced-3(n1286)IV* |
| WS3687 | *rad-54(ok615)I/hT2 [qIs48] (I;III)* |
| CV842 | *dot-1.1[knu337-(pNU1092-KO loxP::hygR::loxP)]I;* *rad-54(ok615)*I/*hT2 [qIs48] (I;III); ced-3(n1286)IV* |
| CV843 | *rad-54(ok615)*I/*hT2 [qIs48] (I;III); syp-1(me17)V/nt1[unc-?n754]let-?gls50)(IV;V)* |
| CV844 | *rad-54(ok615)*I/*hT2 [qIs48] (I;III); ced-3(n1286)IV; syp-1(me17)V/nt1[unc-?n754]let-?gls50)(IV;V)* |
| CV845 | *dot-1.1[knu337-(pNU1092-KO loxP::hygR::loxP)]I;* *rad-54(ok615)*I/*hT2 [qIs48] (I;III); ced-3(n1286)IV; syp-1(me17)V/nt1[unc-?n754]let-?gls50)(IV;V)* |
