# Supplementary material for: DOT-1.1-dependent H3K79 methylation promotes normal meiotic progression and meiotic checkpoint function in C. elegans
Source: PLoS Genet. 2020 Oct 26;16(10):e1009171. doi: 10.1371/journal.pgen.1009171 (PMC7644094; doi:10.1371/journal.pgen.1009171)
Supplement: S1 Appendix — (DOCX) [file pgen.1009171.s008.docx]

***zfp-1* mutant worms exhibit similar phenotypes to those observed in *dot-1.1* worms**

In order to validate the interchangeable use of *zfp-1* and that this mutant can be equated to *dot-1.1* in our analyses (Fig 6A) we evaluated some of the phenotypes observed in the *dot-1.1; ced-3* worms in the *zfp-1* mutant (S3 and S4 Fig). We first analyzed meiotic progression. SUN-1 (pS8) immunostaining revealed that similar to the observation made for *dot-1.1; ced-3* worms, *zfp-1* mutant worms exhibit an extension in the presence of SUN-1(pS8)-positive nuclei compared to wild-type suggesting problems with meiotic progression (Figure 2C and S3A). Then, we analyzed the levels of homolog pairing for the X-chromosome, visualized by localization of the zinc finger protein HIM-8, and for chromosome V, visualized by FISH with a probe to the 5S rDNA locus. We observed a delay in pairing as shown by the lower levels of nuclei with paired HIM-8/5S rDNA signal starting at transition zone and persisting into early pachytene compared with the wild-type worms (S3 B Fig; zone 3, P< 0.05 for X-chromosome, P<0.0005 for chromosome V, and zone 4, P<0.001 for X-chromosome, P<0.025 for chromosome V, Fisher’s exact test). We also examined SC assembly in *zfp-1* mutants scoring the percentage of nuclei with complete synapsis as a function of meiotic progression. Similar to wild type and *dot-1.1; ced-3* mutants, *zfp-1* mutants initiated SC assembly at transition zone (zone 3, Fig 3A and S3C Fig). However, the percentage of nuclei with complete synapsis by early pachytene is significantly lower compared to wild-type (65.2% vs 88.3%, P<0.001, Fisher’s exact test), but similar compared with *dot-1.1; ced-3* (65.2% vs 65.4%). Furthermore, more detailed analysis showed that 5.8% (10/173) of the nuclei in mid to late pachytene (zones 5 and 6; n= 173) did not have SYP-1 signal on at least one chromosome compared to 0.65% (n=154) observed in wild-type worms. From those, 10% (1/10) also lacked HTP-3 signal explaining the absence of SYP-1 since proper assembly of the SC depends on the normal formation of axes. Co-immunostaining for SYP-1, HTP-3 and HIM-8 revealed that 70% of the chromosomes without SYP-1 signal (7/10) were positive for HIM-8, similar to what we observed for *dot-1.1; ced-3* mutant worms (S3C Fig and Fig 3B).

We evaluated meiotic DSB repair progression by quantifying the levels of RAD-51 foci on whole-mounted gonads in *zfp-1* mutant worms (S4A and S4B Fig). We found that similar to the *dot-1.1; ced-3* mutant worms, *zfp-1* worms showed a decrease in the number of RAD-51 foci in zones 3, 4 and 5 compared to wild type worms (P<0.0003, by the two-tailed Mann-Whitney test, C.I. 95%). Furthermore, when we assessed meiotic DSB progression in *zfp-1; syp-1* double mutants we observed a significant decrease in the number of RAD-51 foci compared to the *syp-1* mutant alone starting at TZ (zone 3) and persisting to late pachytene (zone 7) (P<0.0003, by the two-tailed Mann-Whitney test, C.I. 95%), supporting the role of H3K79me in meiotic checkpoint function.

Finally, we evaluated chromosome morphology defects in diakinesis in *zfp-1* mutant worms. Similar to what has been observed for *dot-1.1; ced-3* mutant worms this analysis revealed 10.8% of oocytes showing aberrant chromosome condensation in *zfp-1* mutant worms compared to 0% in wild type. *zfp-1* mutants also showed low levels of additional chromosome defects including the presence of fragments (2.7% compared to 0% in wild type), and aggregates (2.7% compared to 0% in wild type) (S4C Fig).

In conclusion *zfp-1* mutant worms show similar phenotypes to what we observed for *dot-1.1; ced-3* mutants suggesting that all of these defects are related to H3K79me reduction. Differences in the percentage of defects observed can stem from the nature of the mutants (*dot-1.1* is a null whereas *zfp-1* is not) and is supported by differences in the levels of H3K79me reduction observed comparing *dot-1.1; ced-3* and *zfp-1* mutants (Fig 1).
